# Myelin Surfactant Assemblies as Dynamic Pathways Guiding the Growth of Electrodeposited Copper Dendrites

**DOI:** 10.1021/jacs.4c04346

**Published:** 2024-07-03

**Authors:** José Ferreira, Jeroen Michiels, Marty Herregraven, Peter A. Korevaar

**Affiliations:** † Institute for Molecules and Materials, Radboud University, Heyendaalseweg 135, Nijmegen 6525 AJ, The Netherlands; ‡ TechnoCentre, Faculty of Science, Radboud University, Heyendaalseweg 135, Nijmegen 6525 AJ, The Netherlands

## Abstract

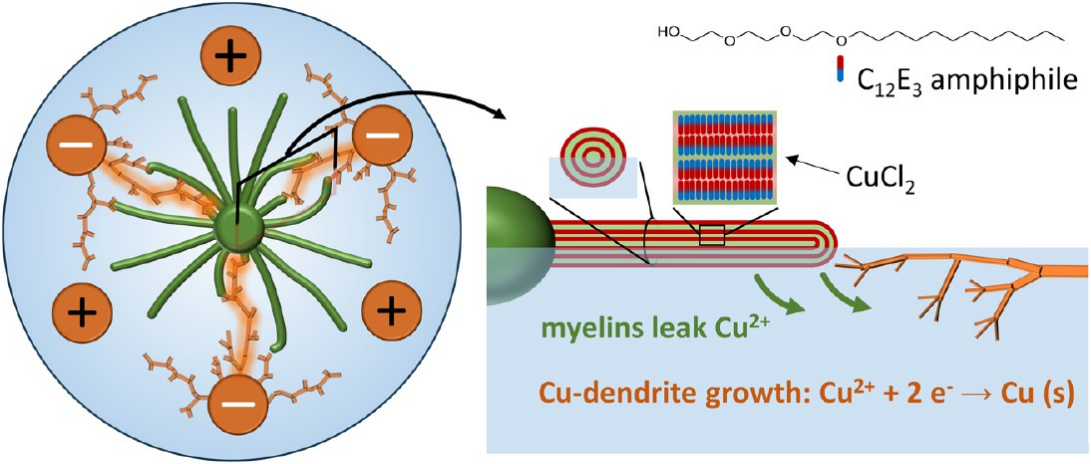

Self-organization of inorganic matter enables bottom-up construction of materials with target shapes suited to their function. Positioning the building blocks in the growth process involves a well-balanced interplay of the reaction and diffusion. Whereas (supra)molecular structures have been used to template such growth processes, we reasoned that molecular assemblies can be employed to actively create concentration gradients that guide the deposition of solid, wire-like structures. The core of our approach comprises the interaction between myelin assemblies that deliver copper(II) ions to the tips of copper dendrites, which in turn grow along the Cu^2+^ gradient upon electrodeposition. First, we successfully include Cu^2+^ ions among amphiphile bilayers in myelin filaments, which grow from tri(ethylene glycol) monododecyl ether (C_12_E_3_) source droplets over air–water interfaces. Second, we characterize the growth of dendritic copper structures upon electrodeposition from a negative electrode at the sub-mM Cu^2+^ concentrations that are anticipated upon release from copper(II)-loaded myelins. Third, we assess the intricate growth of copper dendrites upon electrodeposition, when combined with copper(II)-loaded myelins. The myelins deliver Cu^2+^ at a negative electrode, feeding copper dendrite growth upon electrodeposition. Intriguingly, the copper dendrites follow the Cu^2+^ gradient toward the myelins and grow along them toward the source droplet. We demonstrate the growth of dynamic connections among electrodes and surfactant droplets in reconfigurable setups—featuring a unique interplay between molecular assemblies and inorganic, solid structures.

## 1 Introduction

The emergence of shape is a fascinating feature of living matter.^1,2^ In synthetic settings, also a wide diversity of solid structures with “life-like” appearances can be created via relatively simple experimental protocols. Examples include polycrystalline biomorphic shapes such as micro flowers,^3−7^ corals, and helices that form via coupled precipitation reactions; “chemical gardens” that emerge from combinations of reaction-diffusion, convection, and precipitation,^8,9^ and electrochemically grown dendrites with fractal branches^10,11^ that resemble geometries found in trees, fungi networks, and neurons. Applications of such matter range from optical waveguides^12^ to micropatterned materials^13^ to neuromorphic circuitry.^14,15^

The bottom-up formation of structure strongly relies on a fine balance over physicochemical gradients that guide positioning of the chemical building blocks throughout the growth process.^16^ Such self-organization does not require dedicated (e.g., lithographic) instrumentation for top-down manipulation but critically relies on control over the interplay of reaction, diffusion, and gradients involved. Molecular and supramolecular systems enable a wide diversity of strategies to direct the growth of solid structures.^17^ Self-assembled monolayers or proteins have been used to template crystallization;^18−21^ one-dimensional supramolecular assemblies to cast nanowires from metals or inorganic material;^22−28^ (supra)molecular ligands or DNA strands to couple nanoparticles into materials^29−32^ or functional nanocavities;^33,34^ and proteins to catalyze the metallization of nanowires among electrodes in neuromorphic circuits.^35^ Furthermore, hydrogels and viscous polymer solutions have been exploited to modulate diffusion, reaction, or crystallization dynamics and thereby generate structured hybrid materials.^36−39^

**Figure 1:**
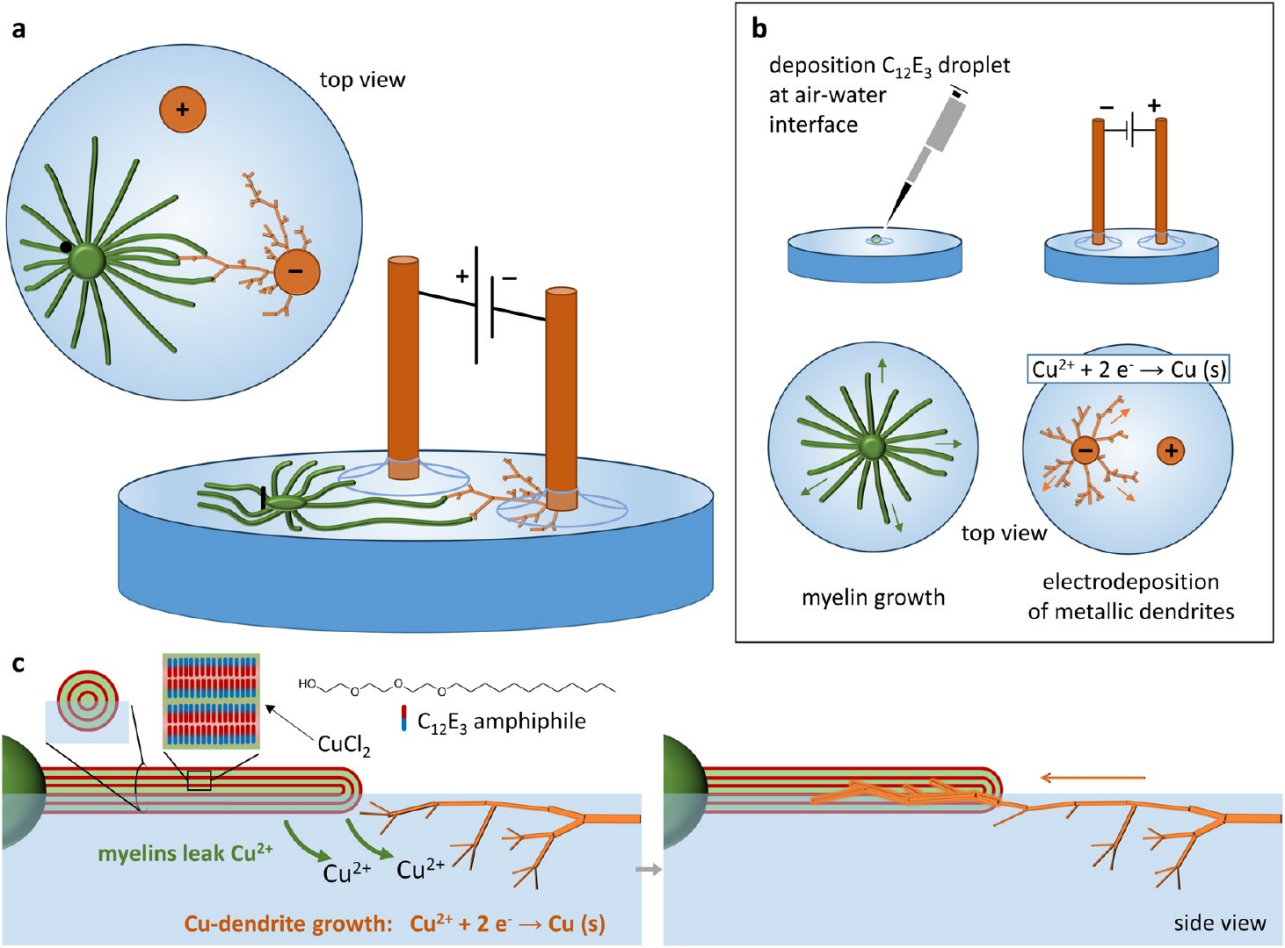
Myelin filaments guiding the growth of electrodeposited copper dendrites at the air–water interfaces. (a) Schematic representation of myelin assemblies (green) that grow over an air–water interface and deliver copper(II) (Cu^2+^) ions, which are electrodeposited upon reduction and result in copper dendrites that grow from a negative electrode. (b) As building blocks of our system, we exploit (1) myelin filaments, growing from a droplet of C_12_E_3_ amphiphiles when deposited at the air–water interface and (2) the dendritic, fractal structures that grow from the surface of a negative electrode when placed in a solution of Cu^2+^ ions, via the reaction Cu^2+^ + 2 e^→^ → Cu (s). (c) When a copper(II) salt is loaded in the bilayer structure of the tri(ethylene glycol) monododecyl (C_12_E_3_)-based myelins, Cu^2+^ ions leak from the myelins in the surrounding aqueous solution and thereby create a Cu^2+^ gradient that directs the growth of the copper dendrites upon electrodeposition–ultimately allowing the dendrites to grow along the copper(II)-loaded myelins.

**Figure 2:**
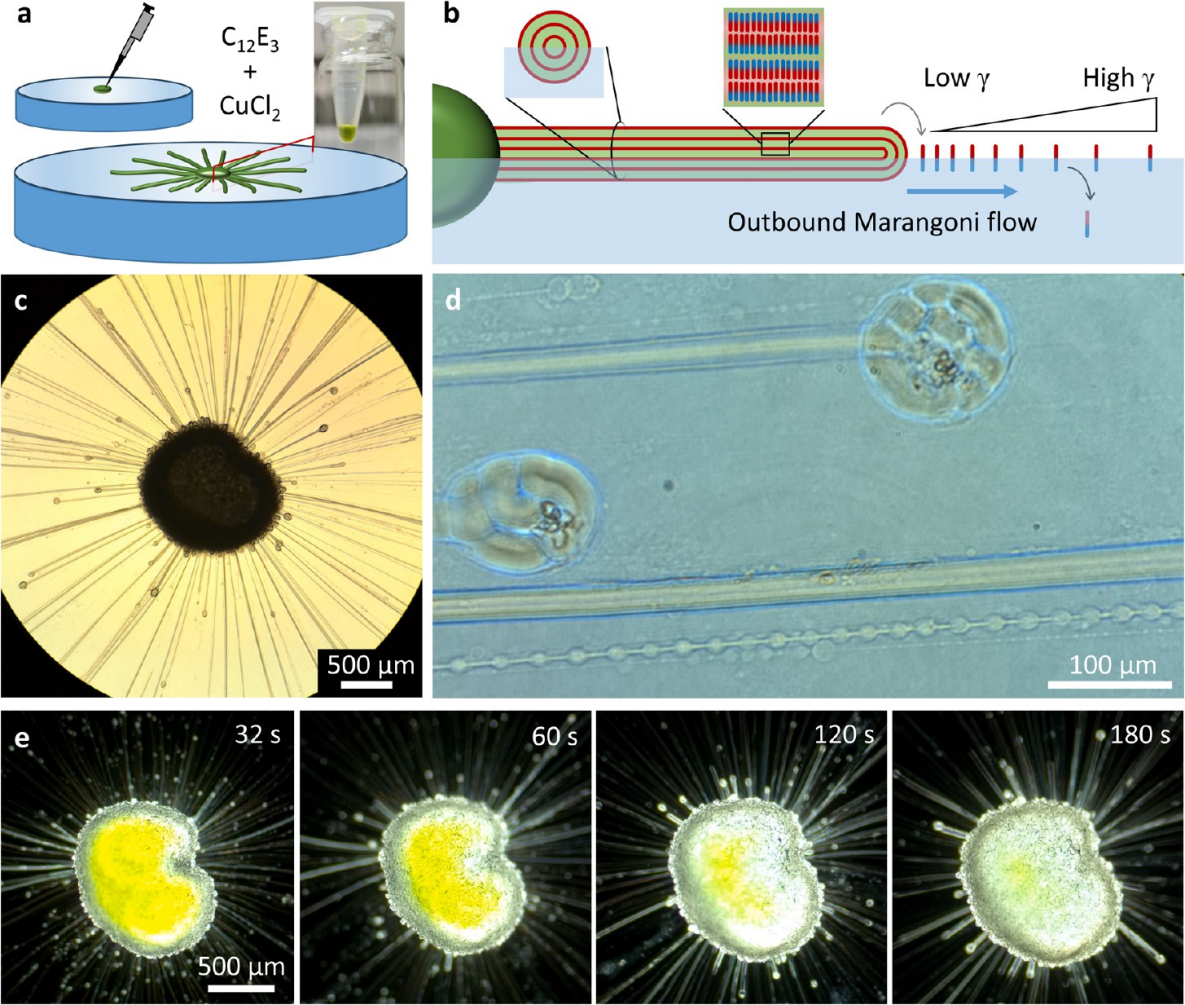
Growth of C_12_E_3_-based myelins loaded with CuCl_2_ over the air–water interface. (a) Myelins grow from a microliter CuCl_2_/C_12_E_3_ source droplet, a green/yellow liquid, as shown in the photograph of the Eppendorf tube, when deposited at the interface of milli-Q water. (b) The myelin growth is driven by the release of C_12_E_3_ surfactants to the air–water interface, which subsequently dissolve in the underlying aqueous solution, generating a surface tension (γ) gradient that drives the outbound Marangoni flow along the air–water interface that extrudes the myelins from the droplet. (c) Optical microscopy image of the myelins, growing from a source droplet (1.0 μL, 16.7 mol % CuCl_2_). (d) The inclusion of CuCl_2_ affects the myelin structure, generating spheroids at their tips, whereas the thinnest myelins show pearling. (e) Optical microscopy recording of the infusion of water into the CuCl_2_-loaded source droplet, displaying a color change from green-yellow to light blue, indicative of copper(II) hydration.

Here, we establish a dynamic molecular system that exploits the growth of myelin assemblies floating over an aqueous medium to guide the electrodeposition of copper dendrites by the transport of copper(II) (Cu^2+^) ions (Figure 1a). Without the guiding element, the reduction of copper(II) from an aqueous solution results in a radial growth of dendritic structures from the surface of the cathode^40−45^ (Figure 1b). Due to their conductivity, these wires are repelled from the negative electrode and grow toward the Cu^2+^-rich medium upon electrodeposition at their tips–a combination of reaction, diffusion, and electrostatics that provides these so-called dendrites with a fractal-like morphology. We reasoned that the growth of these copper-based dendrites can be directed by soft and dynamic assemblies that locally release Cu^2+^ ions. To this end, we employ myelins assembled from the amphiphile tri(ethylene glycol) monododecyl ether (C_12_E_3_), loaded with a copper(II) salt [copper(II)chloride, CuCl_2_]. Earlier, we demonstrated how C_12_E_3_ forms a lamellar phase of closely packed bilayers at the boundary of a C_12_E_3_ droplet when deposited at an air–water interface.^46,47^ Swelling of the bilayer phase upon uptake of water drives the growth of multilamellar myelin filaments from the droplet,^48,49^ which spread over the air–water interface. The concomitant release of individual C_12_E_3_ amphiphiles as a surfactant to the air–water interface generates outbound Marangoni flows which extrude myelins from the C_12_E_3_ droplet.^50^ Marangoni flows at the air–water interface are typically directed from regions of low toward high surface tension γ, with high and low surfactant adsorption to the air–water interface, respectively (Figure 2b). As the C_12_E_3_ surfactants are released from the source droplet and subsequently depleted to the bulk phase of the aqueous solution, the outbound Marangoni flow is sustained over the course of the myelin growth experiments. Together, our copper(II)-loaded myelins are anticipated to create localized Cu^2+^ gradients that direct the structures obtained upon copper electrodeposition (Figure 1c).

As the building blocks for our system, we first studied the growth of C_12_E_3_-based myelins loaded with a copper(II) salt in the interior of the lamellar phase. Second, we assess the growth of copper dendrites via electrodeposition under sub-mM conditions–matching the concentration levels of Cu^2+^ anticipated upon release from the myelins. Third, we show how the myelins deliver Cu^2+^ ions at the growing tips of the copper dendrites and thereby direct the path of electrodeposited connections grown in the system. Finally, we explore the potential of the copper(II)-loaded myelins to establish dynamic, reconfigurable connections stemming from a central C_12_E_3_ droplet toward multiple electrodes–a first step toward interconnected networks of electrodeposited wires.

## 2 Results and Discussion

### 2.1 Growth of Amphiphile Myelins Loaded with Cu^2+^ Ions

To grow myelins that are loaded with copper(II) ions, we first mixed copper(II) chloride (CuCl_2_) with the C_12_E_3_ amphiphile. At the highest copper(II) loading of 18.5 mol % (i.e., CuCl_2_/C_12_E_3_ 0.185/1.00 mol) that was used in this study, we observed the CuCl_2_ salt to be entirely dispersed in the liquid C_12_E_3_ phase, forming a homogeneous liquid with a green/yellow color (Figure S1). We tested the filament growth by depositing CuCl_2_/C_12_E_3_ “source” droplets (1.0 μL) on milli-Q water (5.5 mL, 38 mm diameter Petri dish) with traces of C_12_E_3_ at the interface (Figure 2a). The presence of C_12_E_3_ at the air–water interface decreases the surface tension to approximately 28 mN m^–1^, which avoids the source droplet to be exposed to large-surface tension gradients that rapidly tear apart the droplet upon deposition.^51^ Next, the release of C_12_E_3_ from the droplet and filaments to the air–water interface and its desorption from the interface to the bulk solution sustain the outbound Marangoni flow (Figure 2b). As shown in Figure 2c,d, myelins grow from the source droplet–comparable to results obtained with C_12_E_3_-based droplets we reported earlier.^47,50,51^ Furthermore, after deposition, the CuCl_2_/C_12_E_3_ droplets show a gradual color change from dark yellow-green to clear blue, starting from the boundary of the droplet and penetrating the core over a time course of 2 min (Figure 2e and Movie S1). As copper(II) solutions in water are typically blue, even at high concentration, this color change is indicative of the hydration of copper(II) ions upon water intake among the amphiphile bilayers that is inherent to the myelin formation.

Inclusion of CuCl_2_ affects the myelin growth, although their final length (up to 3 mm, Figure S1) is comparable to that of myelins grown from pure C_12_E_3_ source droplets. We observe the formation of spheroids at the tips of the myelins as well as pearling along the thinnest ones (Figure 2d). These morphological features do not occur in the absence of Cu^2+^ (Figure S1), and we hypothesize that those are related to an enhanced interfacial tension of the myelin–water interface due to the interaction between Cu^2+^ and C_12_E_3_. Therefore, we conclude that upon myelin growth from the copper(II)-loaded C_12_E_3_ droplet, the Cu^2+^ ions are at least partly residing in the lamellar phase of the myelin. At the same time, however, Cu^2+^ ions are released to the surrounding aqueous phase, giving an enhanced conductivity of the aqueous solution (vide infra). Together, our results show the growth of copper(II)-loaded myelins, from which the release of Cu^2+^ ions potentially allows for directed electrodeposition of copper dendrites.

### 2.2 Electrochemical Growth of Copper Dendrites

We anticipated that the release of Cu^2+^ from our myelins results in sub-mM concentrations of the copper(II) supply for the growth of the dendrites. For example, upon complete release of Cu^2+^ to the aqueous phase from a 2 μL source droplet containing 18.5 mol % CuCl_2_, the upper limit to the copper(II) concentration in our studies equals approximately 200 μM. This concentration is well below the copper(II) concentrations that are typically used for electrodeposition studies of dense copper fractals.^43−45^ Therefore, we first study the electrochemical deposition of copper dendrites from aqueous CuCl_2_ solutions with concentrations between 10 μM and 1 mM.

**Figure 3:**
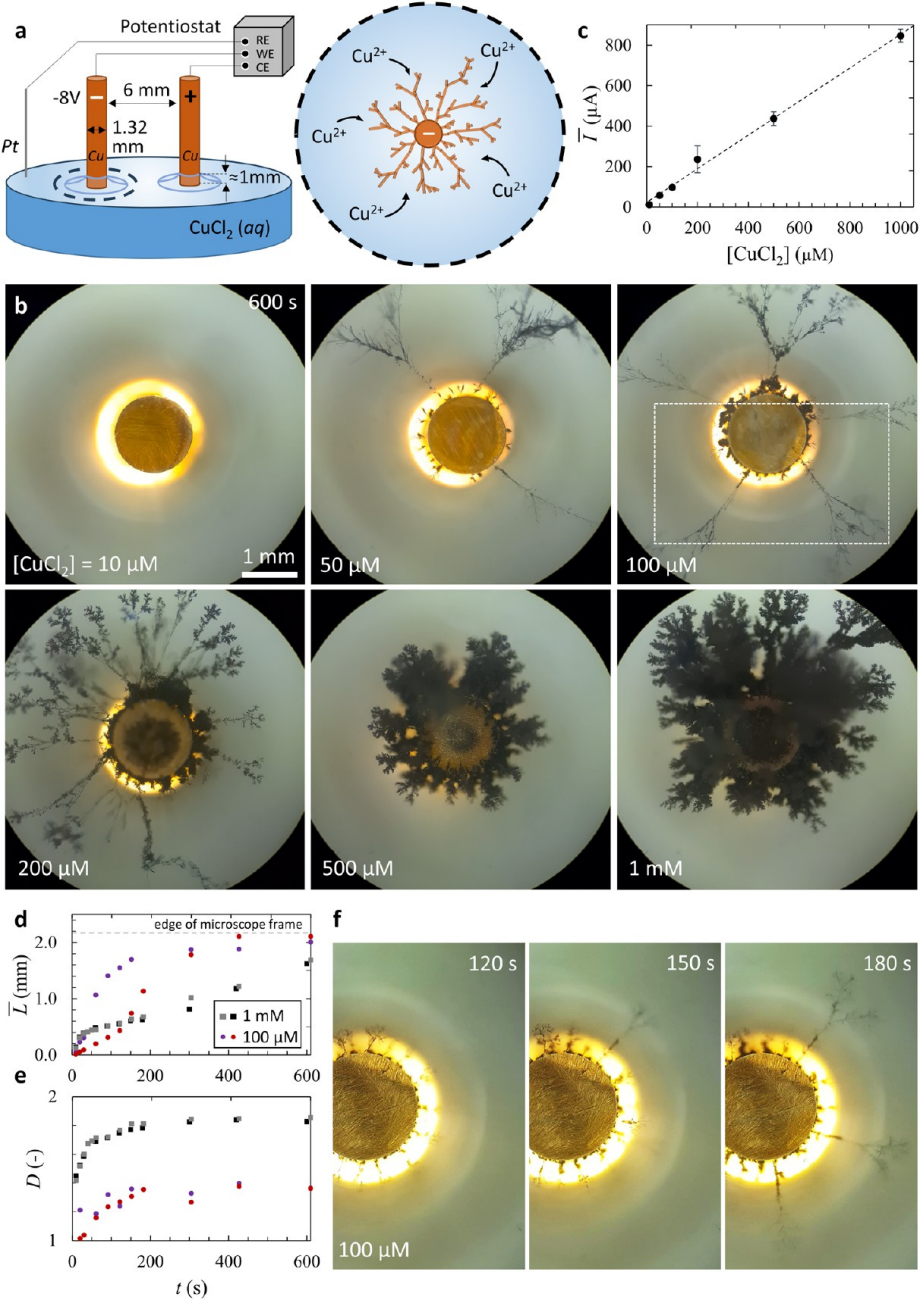
Characterization of copper dendrite growth from CuCl_2_ solutions in the sub-mM concentration regime. (a) Schematic representation of the electrochemical cell, comprising two copper electrodes placed in an aqueous CuCl_2_ solution and connected to a Pt-wire-referenced potentiostat that applies a potential of −8 V to the working electrode. As Cu^2+^ ions are electrodeposited at the negative working electrode, fractal copper dendrites grow from the surface of the electrode toward the surrounding aqueous solution. (b) Optical microscopy images of copper dendrites grown over a time period of 600 s from the negative electrode from solutions with a starting CuCl_2_ concentration ranging from 10 μM to 1 mM. (c) Average current *I* during the electrodeposition vs starting CuCl_2_ concentration in the aqueous solution, based on the first 600 s of the time courses shown in Figure S2. (d) Time-dependent average length *L*, measured from the surface of the electrode, of copper dendrites that are electrodeposited from CuCl_2_ solutions of 100 μM (*n* = 2) and 1 mM (*n* = 2). (e) Time-dependent fractal dimension *D* of copper dendrites that are electrodeposited from CuCl_2_ solutions of 100 μM (*n* = 2) and 1 mM (*n* = 2). (f) Optical microscopy images showing the time-dependent development of the copper dendrites growing from a 100 μM CuCl_2_ solution. The field of view corresponds to the dashed white box in (b). To improve the visibility of the dendrites in the onset of their growth, the contrast of the images in (f) is enhanced.

We used an electrochemical workstation connected to two copper rods, respectively, the working (−) and counter (+) electrode, and a platinum wire as pseudo reference. The electrodes are dipped approximately 1 mm in the aqueous CuCl_2_ solution, such that the copper dendrites that grow from the working electrode stay close to the air–water interface (Figure 3a). By applying a potential of −8 V at the working electrode, dendrite growth is followed over time by optical microscopy (Movie S2). Figure 3b shows how more abundant and denser dendritic structures grow from the working electrode with an increasing copper(II) concentration in the aqueous solution. With 10 μM CuCl_2_, no dendrites were obtained even after 20 min, whereas all solutions with higher CuCl_2_ concentrations (50 μM and higher) did result in dendrite growth, suggesting a threshold concentration required for the nucleation of the dendrites. In Figure 3f, we follow the formation of the copper dendrites over time with a solution of 100 μM CuCl_2_. Up to *t* = 120 s, only tiny structures grow from the electrode surface, whereas around *t* = 150 s, the first elongated dendrite forms. Even though the time point at which the first elongated structure appears is variable, the average dendrite length *L* vs time plot (Figure 3d) shows the growth of elongated structures at 100 μM. Such elongated structures are only formed at intermediate CuCl_2_ concentrations of 50–200 μM (Figures 3b and S2). At higher concentrations (500 μM and 1 mM), the dendrites grow steadily into more densely packed structures directly when the voltage is applied (Movie S2). Whereas these dendrites stay shorter (Figure 3d), their denser packing in comparison to the dendrites obtained at lower concentrations is evidenced by their higher fractal dimension *D* that we obtained using the “Fractal Box Count” plug-in in ImageJ (Figure 3e).^52^ We rationalize the thin dendrites obtained at low CuCl_2_ concentrations by the limited availability of Cu^2+^ ions close to the working electrode. As a consequence, when the aqueous solution closer to the electrode has been depleted by the growing dendrites in the initial phase, sustaining the electrodeposition process requires the dendrites to penetrate further into the aqueous solution for supply of Cu^2+^ ions. In contrast, at higher CuCl_2_ concentrations, the higher supply of Cu^2+^ upon diffusion from the aqueous solution allows electrodeposition to take place closer to the working electrode.

Importantly, no bubbles were observed at the electrodes, suggesting that electrons delivered at the working electrode are predominantly involved in the electrodeposition of copper [i.e., Cu^2+^ (aq) + 2 e^→^ → Cu (s)] rather than in splitting water. The average current intensity increases linearly with the initial CuCl_2_ concentration (Figure 3c), which implies that the rate of copper deposition is proportional to the availability of Cu^2+^ ions in the aqueous solution. The copper deposition (*m*, in mol) can be estimated based on the current intensity *I*(*t*) via , with *z* = 2 and Faraday constant *F* = 96485 C mol^–1^. For example, for the dendrites grown from the 100 μM CuCl_2_ solution, the copper deposition equals approximately 3.1·10^–7^ mol over a time period of 10 min, which represents 56% of the initial copper(II) amount present in the solution. As the current intensity was observed to stay relatively constant (Figure S2), this implies that the deposited Cu^2+^ ions are replaced by new Cu^2+^ ions formed upon oxidation at the copper counter electrode.

### 2.3 Myelins Guiding the Copper Dendrite Growth

**Figure 4:**
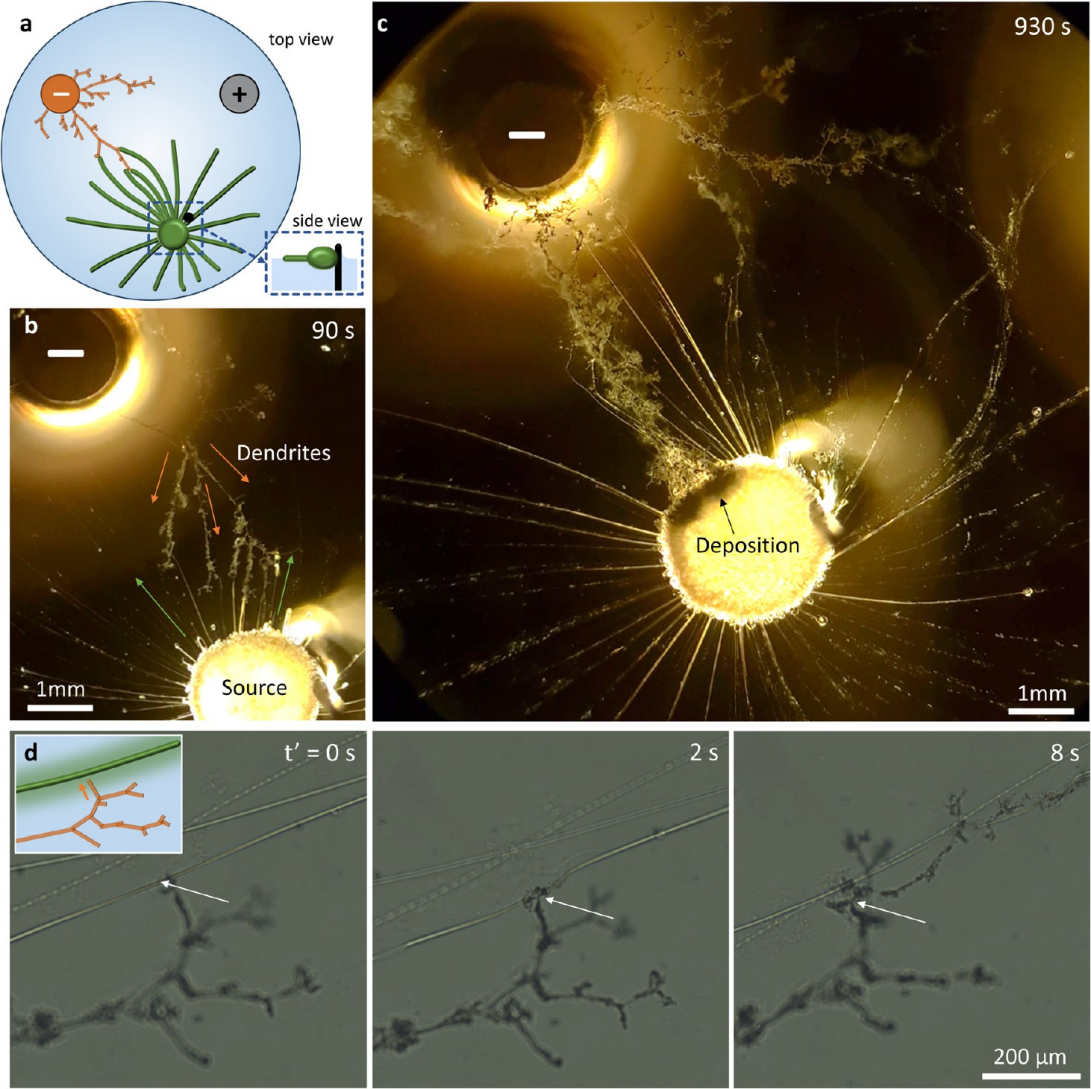
Probing the interaction between the copper dendrites and copper(II)-loaded myelins. (a) Schematic representation of the experiment: In an electrochemical cell, as shown in Figure 3a, with a copper working electrode and a glassy carbon counter electrode, a source droplet loaded with CuCl_2_ (16.7 mol %, 1.0 μL) was placed at the air–water interface and kept in place by the meniscus of a metal pin in the solution at *t* = −208 s. Next, a voltage of −8 V was applied (*t* = 0 s). (b) Optical microscopy image of copper dendrites (orange arrows) growing from the working electrode and interacting with the myelins (green arrows) growing from the source. (c) The dendrites ultimately reach the source droplet, and electrodeposition of copper starts off inside the source droplet. (d) High-magnification optical microscopy recording of copper dendrites that interact and grow along the copper(II)-loaded myelins.

To study the interaction between the copper(II)-loaded myelins and the dendrites that grow from the working electrode, we introduce a C_12_E_3_ source droplet with CuCl_2_ (16.7 mol %) in the electrochemical cell, as shown in Figure 4a and Movie S3. The source droplet is kept in position by tethering it to the meniscus of a metal pin, using the “Cheerios” effect,^53^ which exploits the capillary attraction emerging from overlap in the air–water menisci of the metal pin and the floating source droplet (Figure 4a). Typically, we allow the droplet to settle and start growing myelins for a couple of minutes prior to turning on the potential at *t* = 0 s. When a potential of −8 V is applied, copper dendrites grow from the working electrode within 30 s and tether to the myelins that grow in the direction of the electrode. Upon touching the myelins, the dendrites grow along them by consuming the copper(II) these structures contain (Figure 4d and Movie S3). Thereby, after 90 s, the dendrites span a distance of up to 3.5 mm from the electrode (Figure 4b), featuring a much faster growth compared with the electrodeposition in the absence of the myelins (Figure 3). In the early stages of the growth process, the connection of dendrites and myelins appears to be fragile: some dendrites are pushed away from the source droplet due to the outbound Marangoni flow; disconnect from the myelins; or break from their contact point at the electrode. Over time, however, copper dendrites reach the source droplet while following the filaments (*t* = 464 s, Movie S3). Next, electrodeposition of copper starts inside the source droplet, which transforms the appearance of the source droplet in optical microscopy from yellow to black (Figure 4c). After the dendrites have reached the source droplet, the electrodeposited connection among the working electrode and the source becomes more stable and less sensitive to disturbing Marangoni flows (Movie S3). Together, our results show that the conductive nature of the copper dendrites, evidenced from the electrodeposition taking place at their tips and ultimately in the source droplet, is maintained in the presence of the C_12_E_3_-based myelins. The remaining myelins that have guided the growth of the dendrites upon electrodeposition of their copper(II)-content appear in dark-field microscopy as blurry structures around the dendrites (Figures 4c and S3).

**Figure 5:**
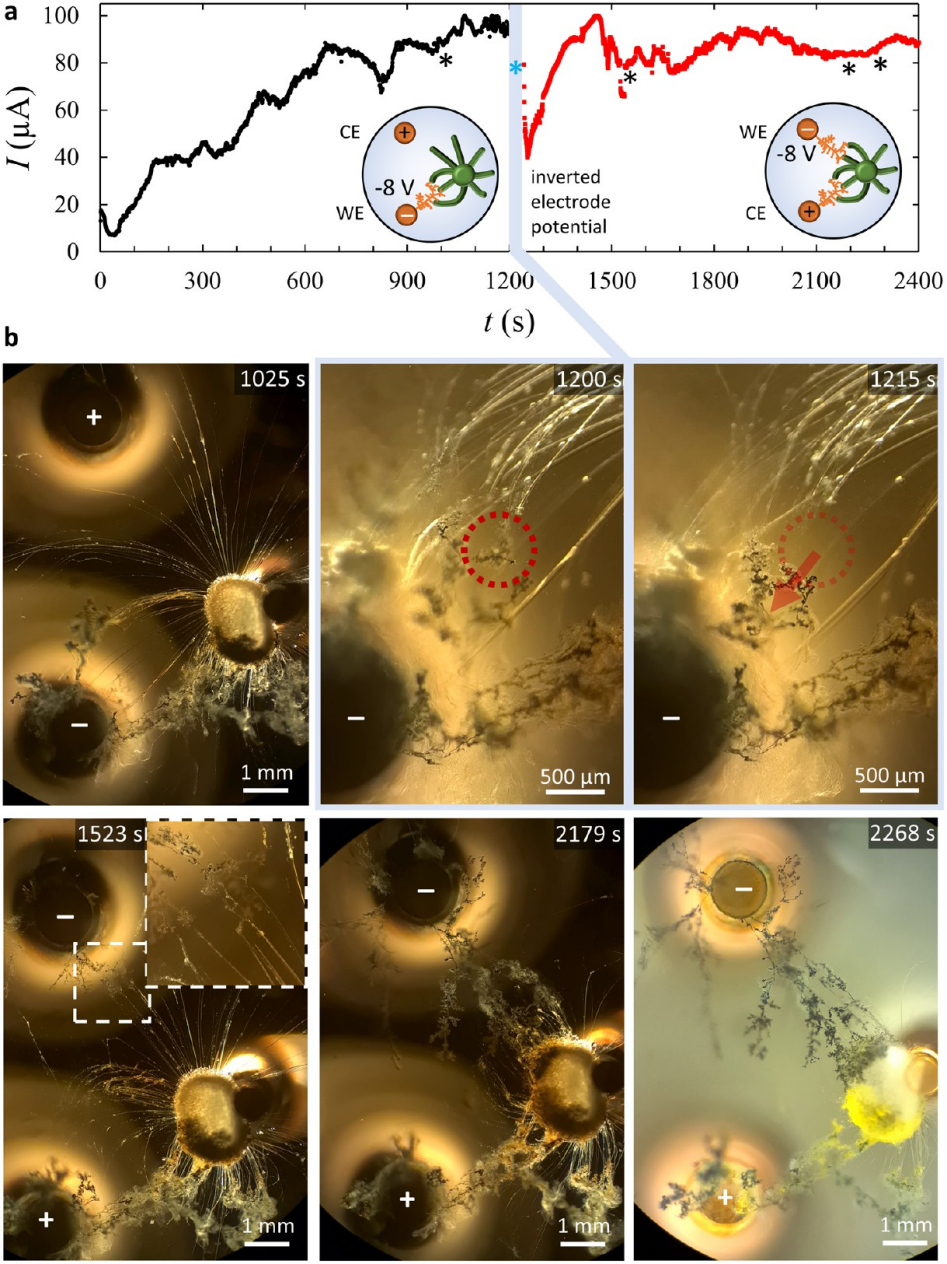
Probing the current involved in the electrodeposition of copper dendrites along copper(II)-loaded myelins. (a) Current *I* vs time for an electrochemical cell, as shown in Figure 3a, with two copper electrodes and a copper(II)-loaded source droplet (1.0 μL, 16.7 mol % CuCl_2_) placed on water at *t* = −47 s. At *t* = 0 s, a voltage of −8 V was applied to the working electrode (bottom), and at *t* = 1200 s, the potential to the electrodes was inverted such that the top electrode became the working electrode. (b) Optical microscopy images acquired at different time points, featuring the electrodeposition of copper dendrites in the source droplet (1025 s); the sudden loss of interaction between dendrites and myelins when the potential is turned off (1200 vs 1215 s); the growth of new dendrites from the top electrode when a negative voltage is applied (1523 s), followed by electrodeposition from this electrode in the source droplet (2179 s); and the color differences between the dendrites electrodeposited in the two cycles, shown in bright-field microscopy (2268 s).

To further unravel the interaction between the dendrites and the myelins, we probe the current over time while following the electrodeposition with optical microscopy—using a similar experimental setup, as shown in Figure 4a, with two copper electrodes (Figure 5a and Movie S4). After deposition of the CuCl_2_-loaded source droplet, a voltage of −8 V is applied at the working electrode, generating a current of approximately 20 μA. This current is well above the conductivity obtained with pure milli-Q water in the same setup (2.6 μA, Figure S2) and indicative of ions being released from the source droplet to the aqueous solution. Over a time course of approximately 100 s, the current first decreases and then increases at *t* = 55 s, concomitant with the sudden growth of a single dendrite branch from the working electrode that establishes the first connections with the myelins. We ascribe the initial decrease in the current to the delay in the growth of myelins: the Cu^2+^ ions released initially are consumed upon electrodeposition, such that the current declines first. As sufficient myelins growing from the source arrive close to the working electrode, Cu^2+^ ions are supplied at sufficient concentrations to the growth front of the dendrites, increasing the rate of electrodeposition again. Indeed, in other experiments where the potential was applied later, such that myelins had more time to develop prior to the onset of the electrodeposition, the initial decrease in the current was not observed (Figure S4).

As the dendrites grow and interact with myelins, the current steadily increases. At 416 s, the first dendrites that followed the myelins reach the source droplet, where electrodeposition of copper starts. Around *t* = 900 s, the current levels off at approximately 90 μA, establishing an electrodeposition rate equivalent to the deposition of copper dendrites from a 100 μM CuCl_2_ solution (Figure 3c), even though this exceeds the maximum concentration upon full release of Cu^2+^ from the source (95 μM). This implies that the electrodeposition rate is enhanced by the local availability of Cu^2+^ at a high concentration in the myelins. Furthermore, during the electrodeposition of dendrites that grow from Cu^2+^ ions delivered by the myelins, the current increases from approximately 20–100 μA (Figure 5a). This increase in the current indicates that the rate of electrodeposition increases over time as the dendrites establish more connections with the myelins—and ultimately the source droplet—that provide the copper(II) supply at localized, high concentrations.

We note that the dynamic interaction between dendrites and myelins relies on the counteracting roles of the Marangoni flow, which concomitantly “pushes off” the growing dendrites from the source droplet, while at the same time transferring the copper(II)-loaded myelins toward the growth front of the dendrites and thereby providing them with a path to grow rapidly “upstream” toward the source droplet. The current intensity fluctuates over time due to this dynamic interplay between dendrites and myelins (Figure 5a) and we ascribe variability in the dendrite–myelin interactions and electrodeposition rates between different experiments to this point as well (Figure S4). Additionally, we hypothesize that the rate of electrodeposition in the source droplet depends on variability in the uptake of water, which affects the diffusion of Cu^2+^ within the source toward the growth front. However, even though the current intensities, as a measure for variation in electrodeposition rate, range from 30 to 160 μA (*n* = 5), our experiments show consistently an increase of current intensity as the dendrite–myelin interactions are established (Figure S4).

The reversibility of the connections that are formed upon electrodeposition was assessed by inverting the potentials applied to the two electrodes at 1200 s, as shown in Figure 5a. Immediately after a negative potential of −8 V is applied to the new working electrode, the current drops to 40 μA over a time course of approximately 10 s, presumably due to the depletion of Cu^2+^ ions that were released close to the electrode in the time period [0–1200 s]. As dendrites nucleate from this electrode and connect to the myelins supplying Cu^2+^ for electrodeposition, the current increases again. Again, the dendrites reach the source droplet as they grow along the myelins at *t* = 1800 s, and the current levels off at approximately 85 μA. Importantly, this current intensity implies that the electrodeposited dendrites do not establish a conductive pathway via the source droplet that connects both electrodes. Indeed, bright-field optical microscopy images reveal that the dendrites as well as the electrodeposited material in the source droplet that have been formed in the time period [0–1200] s have turned (in part) yellow (Figure 5b). This suggests the oxidation of copper, while these structures were in contact with the positive electrode in the time period [1200–2400] s.

Together, our results show that the dendrites have a strong preference to grow toward the myelins and source droplet, as their main supply of Cu^2+^ ions. Furthermore, a negative potential is required to sustain the electrodeposited structures. Despite partly oxidation, which can already take place when exposed to a negative potential,^40^ the conductive nature of the copper dendrites is further demonstrated by their repulsion from the working electrode and their mutual repulsion as they continuously move to avoid each other (Figure 3 and Movie S2). We reason that these repulsion effects cause the dendrites to spread out, which, in turn, enhances their chance to encounter a myelin at the air–water interface, grow along the Cu^2+^ gradient toward the myelin, and consume the Cu^2+^ carried by the myelin. At the same time, the interaction with the myelins keeps the high-density copper dendrites afloat at the air–water interface. For example, we note that when switching the potential from −8 to 0 V at *t* = 1200 s, the dendrites that were growing from the working electrode and tethering to the myelins instantly detached from these myelins as they relaxed backward to the electrode and also sank from the air–water interface (Figure 5b and Movie S4). In contrast, the dendrites that were connected to the source droplet and therefore more embedded in supportive myelin structures remained present at the air–water interface.

### 2.4 Reconfigurable Connections in Electrodeposited Networks

**Figure 6:**
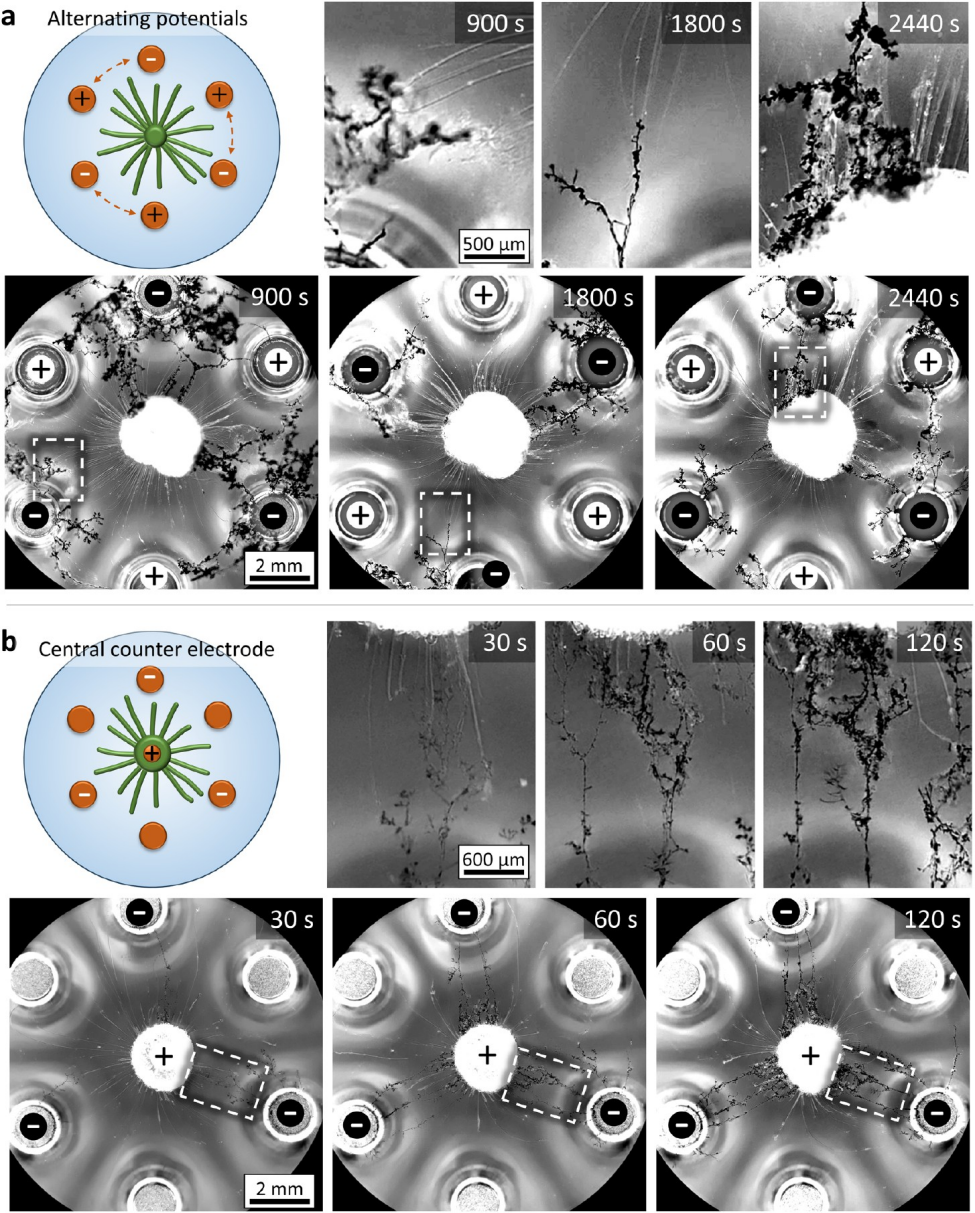
Multiple connections among electrodes and source droplet upon electrodeposition. (a) A copper(II)-loaded source droplet (2.0 μL, 18.5 mol %) is positioned among six copper electrodes in a hexagonal array on water. The source is kept in position via a copper wire inserted in the droplet. The optical microscopy images feature three cycles where in an alternating fashion, three electrodes are the working electrode (−8 V) and three electrodes are the counter electrode. As the potential to the electrodes is switched, we observe the old dendrites to decline and new dendrites to grow from the three working electrodes while interacting with the copper(II)-loaded myelins, some of which reaching the central source droplet. (b) A copper(II)-loaded source droplet (2.0 μL, 18.5 mol %) is positioned among six copper electrodes in a hexagonal array on water. A copper wire is inserted in the source droplet. When a voltage of −8 V is applied to three working electrodes and the copper wire inserted in the source droplet functions as the counter electrode, dendrites grow rapidly toward the source. To improve the visibility of the myelins (20–80 μm diameter) in the bright-field optical microscopy images, the image is converted to grayscale, and the contrast is enhanced.

We explore how copper(II)-loaded myelins allow for the growth of multiple connections, generating dynamic networks of electrodeposited wires. First, we assessed the connections growing via electrodeposition when a source droplet was placed among six copper electrodes positioned in a hexagonal array, as shown in Figure 6a, Movie S5 and Figure S5 (details of the setup). To increase the availability of Cu^2+^ for growing dendrites from multiple electrodes toward the source droplet, we have increased the droplet size to 2.0 μL and the CuCl_2_-loading to 18.5 mol % (Figure S6). With three electrodes as the working electrode (−8 V) and three electrodes as the counter electrode, we observe that the dendrites grow along the myelins, reaching the source at approximately 3 min and maintaining the network over a time course of 15 min during which the voltage was applied. Importantly, the established connections can be reconfigured as upon inverting the applied potential to the electrodes at *t* = 15 min, new dendrites grow from the new working electrodes and start to connect with the myelins, whereas the old dendrites degrade and are removed due to the outbound Marangoni flow from the source droplet. After 15 min, comparable behavior was observed for a third cycle of switching the applied potential to the electrodes, again over a time course of 15 min. In subsequent cycles, myelin growth was observed to decline and the dendrites failed to interact with the myelins that were left. We hypothesize that at this point, most Cu^2+^ ions have leaked from the source and the myelins to the aqueous solution, such that the dendrites cannot grow along a Cu^2+^ gradient toward the remaining myelins anymore.

Even though the dendrites that grow from the three different electrodes typically show interactions with the myelins—establishing a connection from the electrode to the source, not all dendrites grow up to the source droplet itself (Figure 6a). We ascribe the nondirected growth of dendrites to the copper gradients produced upon Cu^2+^ release from the neighboring copper counter electrodes. Furthermore, the dendrite growth strongly depends on the center-to-center distance from the electrodes to the source droplet, as evidenced by the significant increase in electrodeposition when the distance is decreased from 6 to 4 mm (Figure S7). This suggests that more Cu^2+^ ions are delivered by myelins to electrodes positioned closer to the source, resulting in higher electrodeposition rates. Indeed, higher currents were observed for experiments with shorter electrode-source distances (Figure S7). Furthermore, the current increases over time (Figure S7), comparable to the increase in current in experiments with a single electrode (Figures 5a and S4). Combined, our results indicate that delivery of Cu^2+^ ions by the myelins establishes a positive feedback mechanism that favors the growth of dendrites reaching closer to the source: whereas the onset of the electrodeposition relies on Cu^2+^ ions that have been delivered to the (remote) electrode, Cu^2+^ is more abundant to the tips of growing dendrites interacting with myelins closer to the source droplet. In a setting with multiple electrodes, this positive feedback contributes to the nonsymmetric emergence of dendrite–myelin interactions, with variability in the myelin–dendrite interactions as some electrodes establish stronger interactions with the source droplet than others—as we observe in our experiments (Figures 6a and S7).

In a second approach, to avoid the release of copper from the copper counter electrodes, we used gold-plated electrodes to grow dendrites and connect them with a copper(II)-loaded source droplet. In experiments with gold-plated electrodes, typically the dendrites of only one of the three working electrodes reach the source, featuring a “winner-takes-all” scenario: When the first dendrites reach the droplet, the dendrites growing from the “competing” electrodes gradually decline (Figures S8–S10). We hypothesize that once a first conductive connection from electrode to source droplet has been established, electrodeposition can take place more easily via this path-of-least-resistance toward the Cu^2+^-rich source droplet, rather than via the dendrites that are only in contact with the Cu^2+^-poor aqueous medium. Additionally, under these conditions, we observe the formation of bubbles at the electrodes, which is typical for water splitting taking place as a side reaction to the intended Cu^2+^ electrodeposition–corroborating that Cu^2+^ is more abundantly present in the experiments with the copper electrodes producing Cu^2+^ at the positive counter electrodes. Whereas in the experiments with copper electrodes (vide supra), the release of Cu^2+^ ions from the counter electrodes allows the dendrites to be sustained from all electrodes, we ascribe the decline of the dendrites growing from the gold-plated electrodes to the absence of background Cu^2+^ ions in solution. However, including Cu^2+^ in the aqueous solution does not generate electrodeposited connections that reach the source droplet from all working electrodes (Figures S6b,c and S11). Importantly, the myelin–dendrite interaction relies on the supply of Cu^2+^ by the myelins, as with a copper-free source droplet, no interaction among the myelins and the dendrites was observed (Figure S12).

In a third approach, to direct the growth of the dendrites from all working electrodes toward the source droplet, we positioned the copper counter electrode in the source droplet, which is placed inside the hexagonal array of electrodes. We reasoned that both the electric field and the Cu^2+^ gradient produced by the central counter electrode further guide the growth of the dendrites toward the central source. Indeed, we observe that when the potential (−8 V) is applied as shown in Figure 6b, Movie S5 and Figure S14, dendrites growing from all three working electrodes have already reached the source droplet after 60 s, and after 2 min, the connections from the electrodes are reinforced with multiple paths connecting to the source droplet. We note that after 5 min, the source droplet starts to swell, presumably due to the production of Cu^2+^ ions or other side reactions happening at the counter electrode, which disrupts the connection of the dendrites to the source droplet.

**Figure 7:**
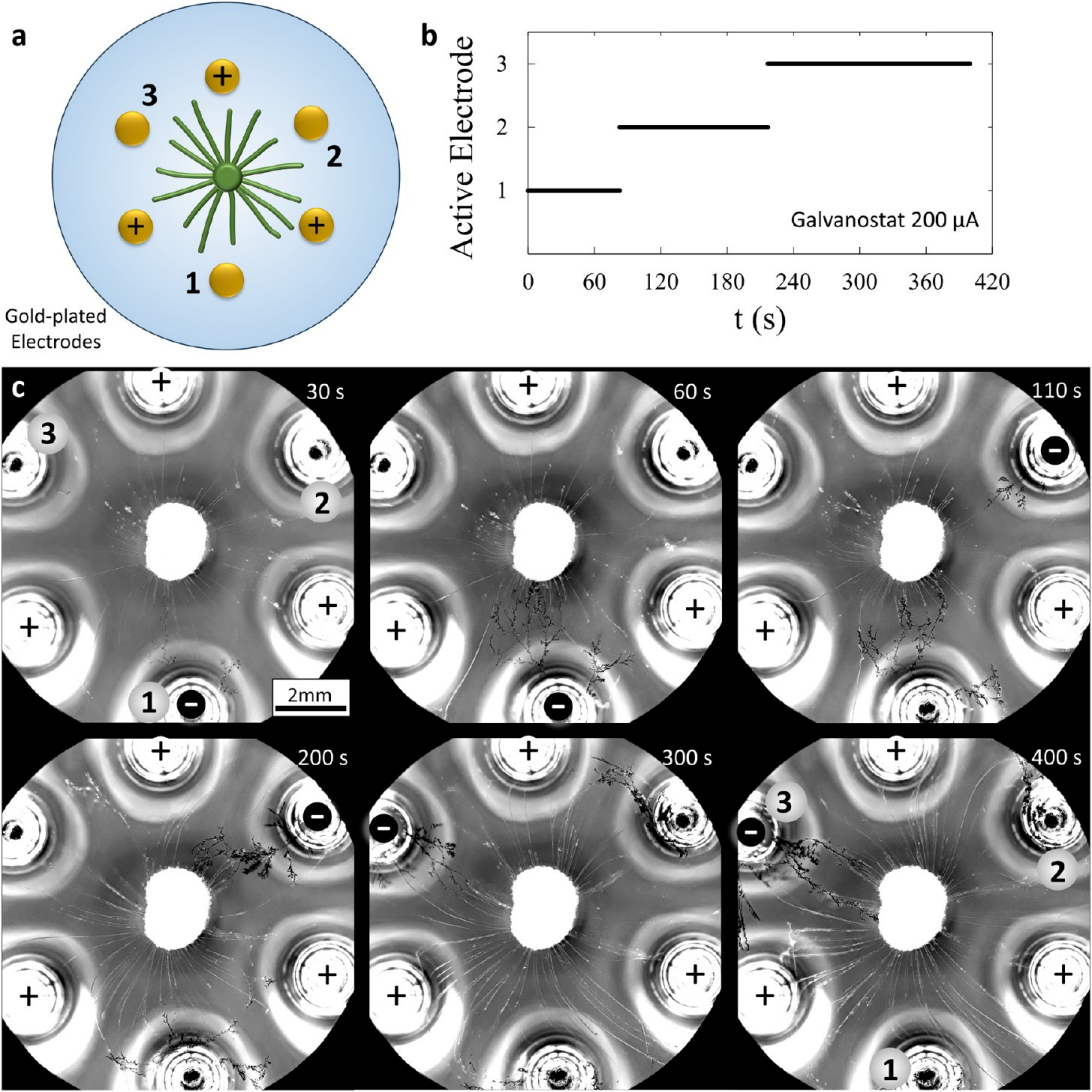
Dynamic, reconfigurable connections among electrodes and source droplets. (a) A copper(II)-loaded source droplet (2 μL, 18.5 mol %) is positioned among six gold-plated electrodes in a hexagonal array on water. The source is kept in position via a copper wire inserted in the droplet. Three electrodes function as the counter electrode, as indicated in the scheme, and the working electrode alternates from positions 1, 2, and 3 over time, as indicated in (b). (c) Optical microscopy images featuring the dendrites that grow from the active working electrode, while the old dendrites from electrodes that are no longer active decline and get pushed away from the source droplet by the Marangoni flow. To improve the visibility of the myelins (20–80 μm diameter) in the bright-field optical microscopy images, the image is converted to grayscale and the contrast is enhanced.

Finally, we establish directional and reconfigurable connections among the electrodes and source droplet, while relying exclusively on the Cu^2+^ gradient produced by the myelins. To this end, we placed a copper(II)-loaded source droplet among six gold-plated electrodes in a hexagonal array (Figure 7, Movie S6 and Figure S13). With three constant counter electrodes and a current limited to 200 μA to suppress bubble formation, we observe that alternating between different working electrodes—one at a time—allows growing dendrites consistently on demand from a specific electrode. In these conditions, we can create connections between a selected electrode and the source droplet. The connection takes between 1 and 2 min to form and features a strong directional character with minimal growth toward the counter electrodes. Furthermore, as the dendrites grow upstream to the outbound Marangoni flow from the source droplet, previously grown dendrites stemming from inactive electrodes are pushed away. Thereby, the Marangoni flow clears the path for myelins to create a new favorable path for the dendrites to grow toward the source when another electrode is activated.

## 3 Conclusions

We demonstrate a unique interaction between myelin assemblies that deliver copper(II) ions to the tips of copper dendrites, which in turn grow along the Cu^2+^ gradient upon electrodeposition. First, we successfully include Cu^2+^ ions among the amphiphile bilayers in the myelins, which grow out from a surfactant droplet over an air–water interface. Next, we show how the myelins deliver Cu^2+^ at a negative electrode placed in solution, allowing the copper dendrites to grow upon electrodeposition. Intriguingly, the growing copper dendrites follow the Cu^2+^ gradient toward the myelins and grow along the Cu^2+^ containing myelins toward the surfactant droplet—displaying a unique interplay between molecular assemblies and inorganic, solid structures. Moreover, we exploit the system to establish reconfigurable connections among electrodes and surfactant droplets via copper(II)-loaded myelins that template the copper electrodeposition. We note that the dendrites can also grow in the absence of myelins toward a source of Cu^2+^ ions, for example, a positive copper electrode or a C_12_E_3_/CuCl_2_ droplet where the myelin growth is inhibited (Figure S15). However, the myelins present a unique growth mechanism as they continuously transfer Cu^2+^ ions from the source droplet to the growth front of the dendrites, allowing for rapid growth of electrodeposited structures. Furthermore, the growth of copper dendrites along the myelins demonstrates the concept of electrodeposition based on building blocks embedded in molecular assemblies that serve as a dynamic pathway to the growth process. Interestingly, our approach—even though based on a simpler system—bears an analogy with the buildup of biominerals in giant marine cells. In *Amphistegina lobifera*, it has been shown how vesicles supply ions, loaded upon endocytosis of seawater, to the calcification site.^54^

With the use of copper-based structures, a negative potential is required to sustain the electrodeposited dendrites, as evidenced by their change in color from black to yellow over a time course of approximately 10 min when the potential to the connecting working electrode is turned off (Figures S8 and S10). As a result, the conductivity of the dendrites—evidenced by the electrodeposition taking place at their tips—is anticipated to decline rapidly when the connections are no longer exposed to a negative potential. In the literature, it has been shown how organic ligands as surface-capping agents increase the stability of copper nanocrystals to oxidation,^55,56^ and we envision that inclusion of such compounds in our myelins can improve the stability of the electrodeposited dendrites. Furthermore, as an alternative to Cu^2+^ ions, the myelin assemblies can be exploited to deliver compounds that produce more stable structures, such as 3,4-ethylenedioxythiophene (EDOT) monomers that form semiconductive polyEDOT connections upon electropolymerization.^14^ We envision that such efforts, due to the general applicability of our concept and its design that only requires simple and easily accessible building blocks, open new routes toward neuromorphic circuits with dynamic, reconfigurable connections emerging from the bottom-up.

## Supporting Information Available

Optical microscopy recording of the CuCl_2_/C_12_E_3_ source droplet deposited on milli-Q water (MP4)
Optical microscopy recordings of copper dendrite growth from working electrodes placed in aqueous CuCl_2_ solutions (10 μM–1 mM) over a time period of 20 min (MP4)
Optical microscopy recordings of copper dendrites growing from the copper working electrode (−8 V) that interact with the copper(II)-loaded myelins growing from the source on milli-Q water (MP4)
Optical microscopy recordings of the interactions of the copper(II)-loaded myelins and the dendrites growing from two different copper electrodes that are consecutively applied as the working electrode (−8 V) (MP4)
Optical microscopy recordings of multiple connections established among a copper(II)-loaded source droplet placed among six copper electrodes in a hexagonal array (MP4)
Optical microscopy recording of dynamic, reconfigurable connections among a copper(II)-loaded source droplet placed among six gold-plated electrodes in a hexagonal array (MP4)
Additional experimental details and materials and methods (PDF)
