# Supplementary material for: Myelin Surfactant Assemblies as Dynamic Pathways Guiding the Growth of Electrodeposited Copper Dendrites
Source: J Am Chem Soc. 2024 Jul 3;146(28):19205–17. doi: 10.1021/jacs.4c04346 (PMC11258786; doi:10.1021/jacs.4c04346)
Supplement: Supplementary file 7 — ja4c04346_si_007.pdf [file ja4c04346_si_007.pdf]

Myelin surfactant assemblies as dynamic pathways guiding the growth of electrodeposited copper dendrites.

José Ferreira[1], Jeroen Michiels[2], Marty Herregraven[2] & Peter A. Korevaar[1,]*

1) Institute for Molecules and Materials, Radboud University, Heyendaalseweg 135, Nijmegen 6525 AJ, The Netherlands
2) TechnoCentre, Faculty of Science, Radboud University, Heyendaalseweg 135, Nijmegen 6525 AJ, The Netherlands
Email: p.korevaar@science.ru.nl

Supplementary Information

Table of contents:

Materials and methods 2

Supplementary Figures 1 – 15 4

Description of Movies 1 – 6 22

Materials and Methods

Materials: The following materials were purchased and used as received from the manufacturers: Tri(ethylene glycol) monododecyl ether (≥98.0%, Santa Cruz Biotechnology Inc., Dallas, TX); copper(II)chloride dihydrate (≥99.0%, Sigma-Aldrich). Copper electrodes are cut from copper wire with 1.5 mm^2 cross-section (Donné Draad B.V., Bergen, The Netherlands). Standard Circular Contacts (16AWG PIN CONTACT) were used as the gold-plated electrodes (1.53 mm diameter, Souriau SAS, Versailles, France). Glassy carbon electrodes (2 mm diameter, type 2) were obtained from Thermo Scientific Chemicals. As pseudo reference electrodes, we used platinum wire (0.0508 mm, 99.95%, Thermo Scientific).

Copper(II)-loaded source solutions: The copper source solutions were prepared by dissolving copper(II)chloride dihydrate in pure tri(ethylene glycol) monododecyl ether ($C_{12}E_3$) upon sonication to achieve a molar ratio of 18.5 mol% (*i.e.* $CuCl_2$:$C_{12}E_3$ 0.185 mol :1.0 mol). The resulting solutions were left to rest for at least 30 min before using. Different molar ratios were then prepared by diluting these solutions with pure $C_{12}E_3$.

Electrode preparation: Right before the experiments, the copper electrodes were buffed with sandpaper of different textures (consecutively P700, P5000 and P7000) to guarantee uniform texture as well as to give the electrode a well-defined cylindrical shape. The gold-plated and glassy carbon electrodes were used without further physical treatment. The electrodes were washed before the experiments with a 1:1 v/v mixture of hexane and isopropyl alcohol, and right before the experiment cleaned first with milli-Q water, then with ethanol and left to dry at ambient conditions.

Studying the interactions amongst copper dendrites and copper(II)-loaded myelins: Optical microscopy images and recordings over time of the self-organization were acquired with an Olympus IX70 inverted microscope equipped with a Samsung S20 ultra mobile phone camera (1440x1440 pixels). ×1.25, x2, x4, x10 and x20 objectives were used to address different morphological features of the dendrites and myelins; brightness and condenser filters were adjusted appropriately to visualize the myelins and dendrites either in brightfield or dark field mode. The optical microscopy recordings were captured at 30 frames per second. The optical microscopy images shown in the Supplementary Movies, as well as the images are unmodified from the raw recording, unless mentioned otherwise – i.e. Figure 3f, Figure 6, Figure 7 and Figure S8. In the latter cases, we increased the contrast of the images to improve the visibility of the myelins (approx. 20 – 80 μm thickness) in wide field microscopy images with a width of approx. 2 cm. The contrast was enhanced using *ImageJ*.

The electrodes were held in position and connected to the electrochemical workstation (*vide infra*) using a homemade device that holds the electrodes and allows to adjust their depth in the aqueous solution as well as their mutual distance (Figure S5). Amperometric and galvanostatic data were acquired using an Autolab PGSTAT20 potentiostat with a platinum electrode as pseudo reference. Optical microscopy recordings and current measurements were acquired simultaneously. In our results, $t = 0$ indicates the moment that the voltage is applied.

To initiate the growth of the myelins, the source droplets were deposited at the air-water interface with a Gilson pipette (0.2 – 2.0 μL range) at the interface of the aqueous medium in a polystyrene Petri dish (lid of a Falcon 35 mm dish, diameter 38.5 mm, height 4.5 mm, filled with 5.5 mL milli-Q water), used as received. To keep the source droplet in position over the course of the experiment, two conditions are used. The source droplet was positioned at the meniscus of a metal pin (folded copper wire, diameter 0.25 mm) that was inserted in the aqueous medium for the experiments shown in Figures 2, 4, 5 (1.0

μL) and S3, S4 (1.0 μL), S6a,c (2.0 μL) and S12 (2.0 μL). For the experiments shown in Figures 6, 7, S6b, S7-11, S13 and S15, the source droplet (2.0 μL) was held in position by 4 twisted copper wires (0.25 mm each) that were inserted in the floating source droplet. These copper wires were guided from the top through a glass Pasteur pipet. The pipet was, in turn, held in place and attached to a micromanipulator (40x40mm XYZ axis linear stage, manually adjustable).

Prior to the deposition of the source droplet at the air-water interface, the surface tension of the medium was decreased by touching the surface of the aqueous medium with a pipet tip dipped in the surfactant sample. This prevents the source droplet from being exposed to large surface tension gradients that rapidly tear apart the droplet upon deposition. We note that the formation of the lamellar phase, required to form the myelins, of the $CuCl_2$/$C_{12}E_3$ source droplets is dependent on temperature. The formation of the laminar phase of $C_{12}E_3$ in our experiments was verified by the opaque appearance of the source droplet to the naked eye after deposition the air-water interface. The experiments shown in Figures 2-5, S6 and S12 were performed at room temperature; experiments shown in Figures 6, 7 and S7-11, S13-15 were also conducted at room temperature but with both source material and medium being kept at 25 ºC prior to the experiments.

Classifying the dendrite growth upon electrodeposition from different $CuCl_2$ concentrations: The average dendrite length *L* was determined from the optical microscopy recordings. Individual frames were analysed in *ImageJ* by encompassing all dendrites stemming from the electrode in the minimum-sized ellipse possible, averaging width and length of the ellipse, subtracting the diameter of the electrode and dividing this value by 2.

The fractal dimension of dendrites *D* was determined with *ImageJ* plug-in "*Fractal Box Count*". First, individual images were converted to 8-bit grayscale, enhanced contrast, and binarized using the *Phansalkar* auto threshold method followed by manual correction of peripheric artifacts and removal of the area referent to the central copper electrode. These images were then analysed by the box counting method with box sizes of 2, 3, 4, 6, 8, 12, 16, 32 and 64. The box counting method counts the number of boxes of an increasing size needed to cover a one-pixel binary object boundary. The box size and the number of boxes necessary to cover the boundary are plotted on a log-log plot and the fractal dimension *D* is determined in the *Fractal Box Count* plug-in from the slope, i.e. *D* = -slope.

Supplementary Figures 1 – 15

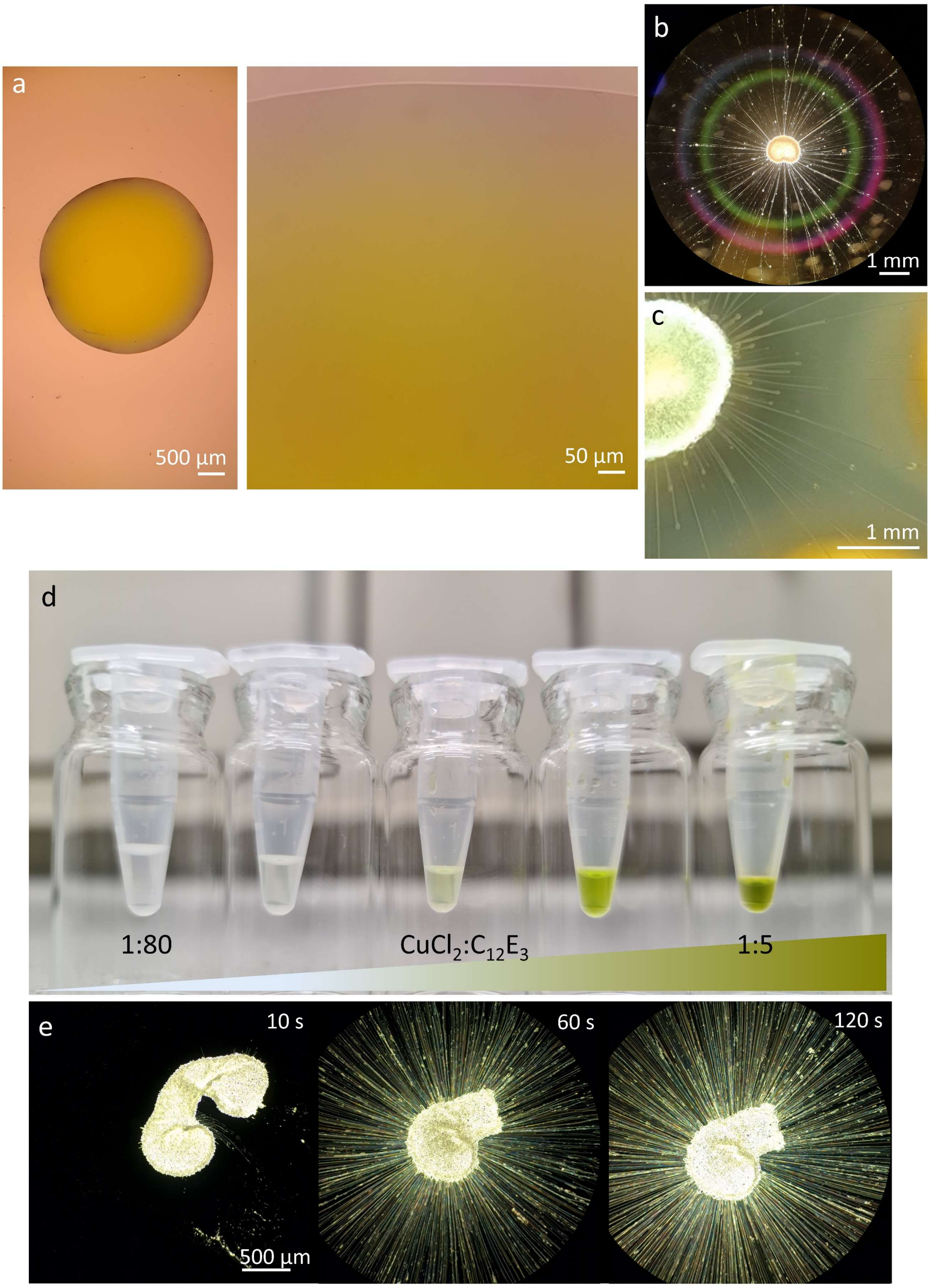

Figure S1: a) Optical microscopy image of $CuCl_2/C_{12}E_3$ source droplet solution (18.5 mol% $CuCl_2$ deposited on a glass slide. No $CuCl_2$ crystals are observed, implying a homogeneous mixture of Cu^{2+} ions throughout the source droplet solution. **b)** Wide field optical microscopy image of myelins growing from a $CuCl_2/C_{12}E_3$ source droplet (16.7 mol% $CuCl_2$, 1.0 µL), deposited on milli-Q water. **c)** Optical microscopy image of myelins growing from a $CuCl_2/C_{12}E_3$ source droplet (18.5 mol% $CuCl_2$, 2.0 µL) deposited on milli-Q water, featuring the spheroids at the tips of the myelins loaded with 18.5 mol% copper(II). **d)** Photograph of $CuCl_2/C_{12}E_3$ source droplet mixtures in Eppendorf tubes: from left to right 1:80, 1:40, 1:20, 1:10 and 1:5 $CuCl_2/C_{12}E_3$ (molar ratio). **e)** Optical microscopy recording of myelins growing from a pure $C_{12}E_3$ source droplet (i.e. without copper(II)-loading) deposited on

milli-Q water. The shape of the droplet, right after deposition is non-spherical. We note that inclusion of $CuCl_2$ in the source droplets enhances their viscosity, which helps to stabilize the source droplets upon deposition. In earlier work, we included sodium alginate in the aqueous medium to enhance the stability of our source droplets. With copper(II)-loaded source droplets, we omitted sodium alginate to avoid complexation with copper(II). Importantly, with copper(II)-free myelins, no spheroids or pearling was observed. Furthermore, these optical microscopy recordings were performed under the same lighting settings as the results shown in Figure 2e, showing that in the absence of Cu^{2+}, no color transition from yellow-green to light blue is obtained in the source droplet.

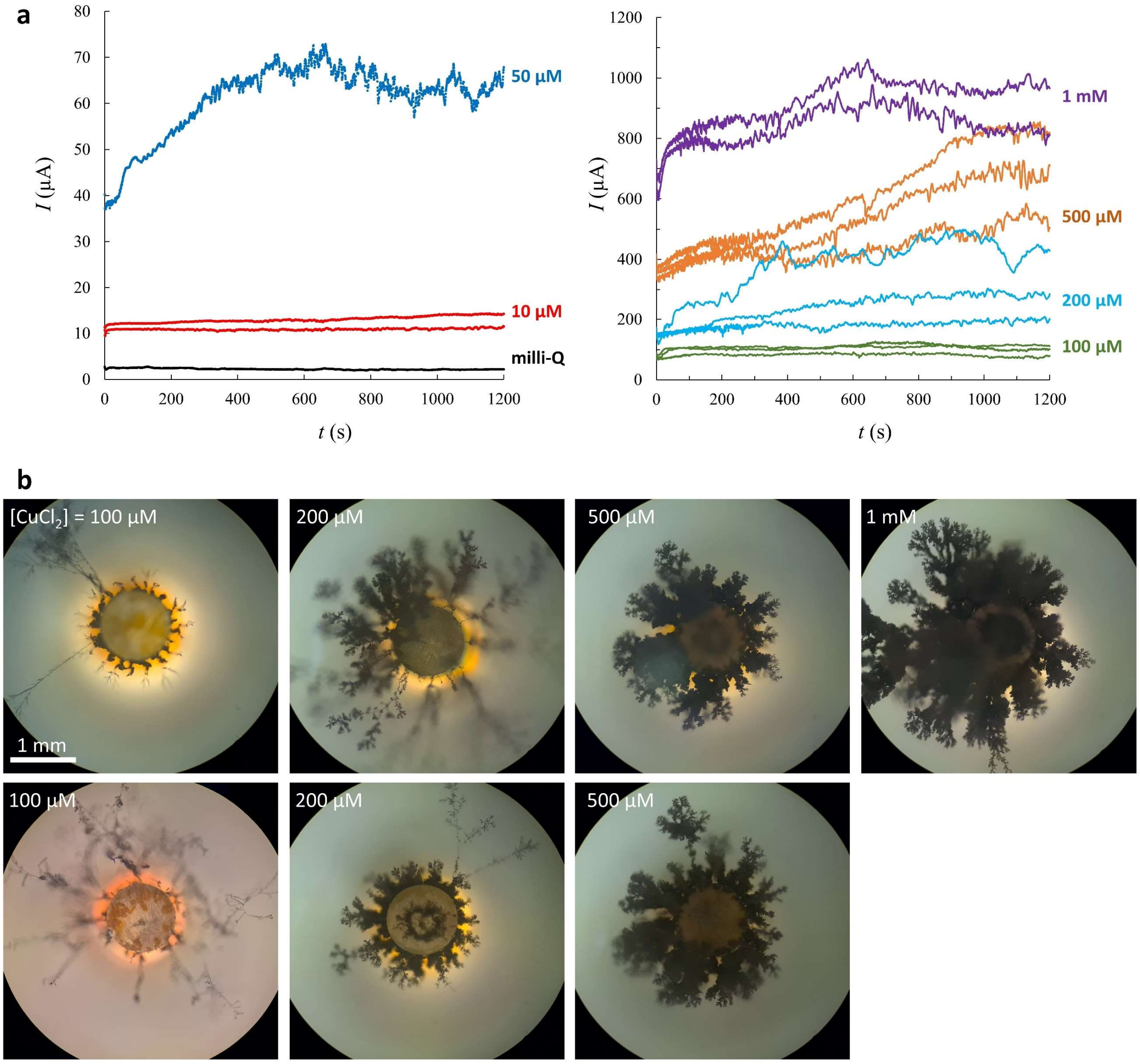

Figure S2: a) Current (*I*) vs time during electrodeposition of copper dendrites from aqueous $CuCl_2$ solutions with different concentrations, acquired using the electrochemical setup shown in Figure 3a and corresponding to the experiments shown in Figure 3b, Figure S2b and Movie 2. The graph of *I* vs time acquired for milli-Q, using the same setup, is shown as well for comparison. **b)** Optical microscopy images of copper dendrites grown over a time period of 600 s from the negative electrode, from solutions with different starting $CuCl_2$ concentrations – complementing the experiments shown in Figure 3b.

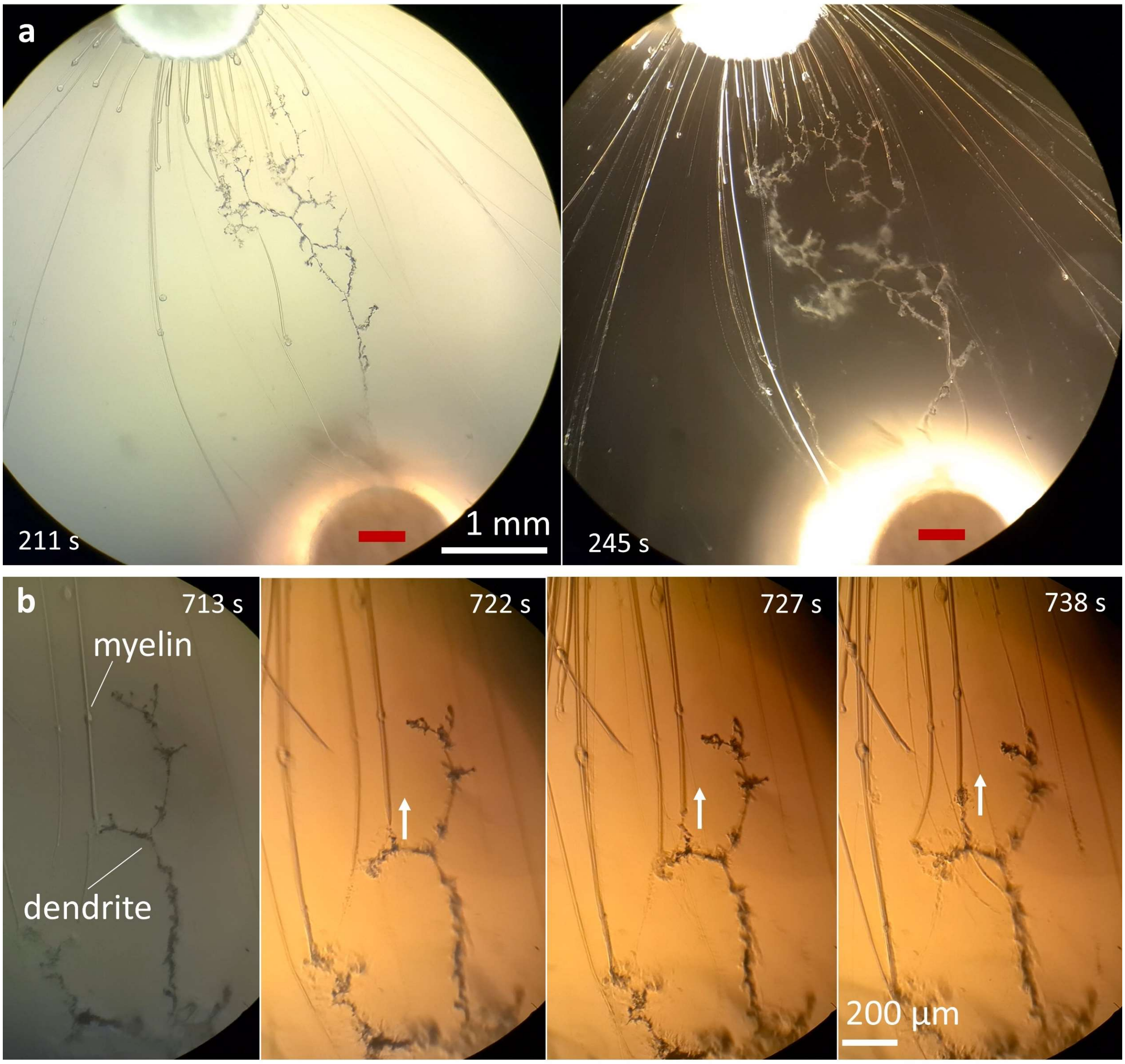

Figure S3: a) Optical microscopy recordings highlighting the interactions with the myelins and dendrites. The source droplet (1.0 µL, 16.7 mol% $CuCl_2$) is deposited on milli-Q water, and the voltage of -8 V is applied to the working electrode at t = 0 s. The image at t = 211 s is recorded in bright field mode and clearly shows the dendrites. The image at t = 245 s is recorded in dark field mode and reveals the myelins as blurry structures covering the dendrites. **b)** Large magnification optical microscopy recordings showing the electrodeposition of the dendrite, progressing over time along the copper(II)-loaded myelin, as indicated by the white arrows.

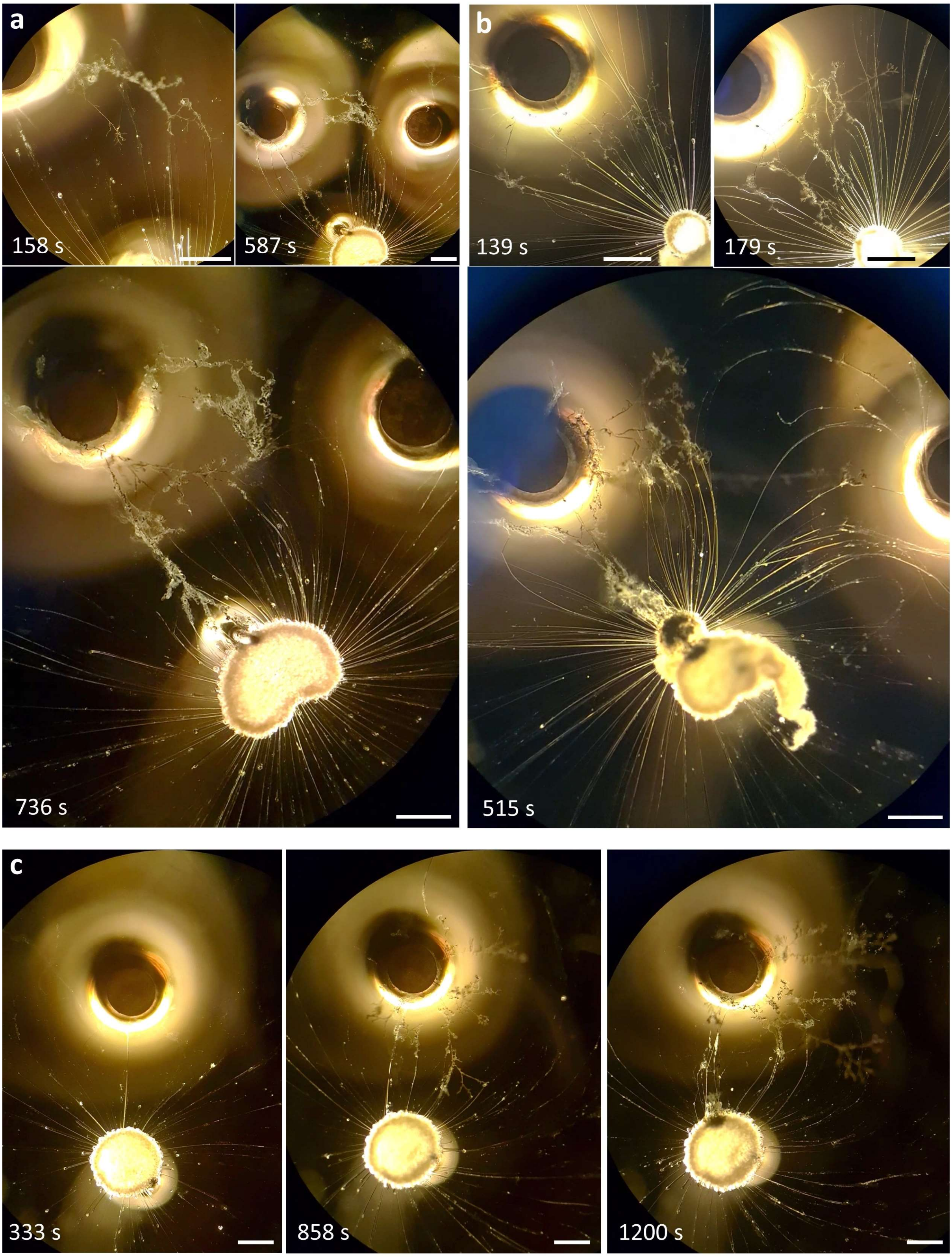

Figure S4 (caption continues on next page)

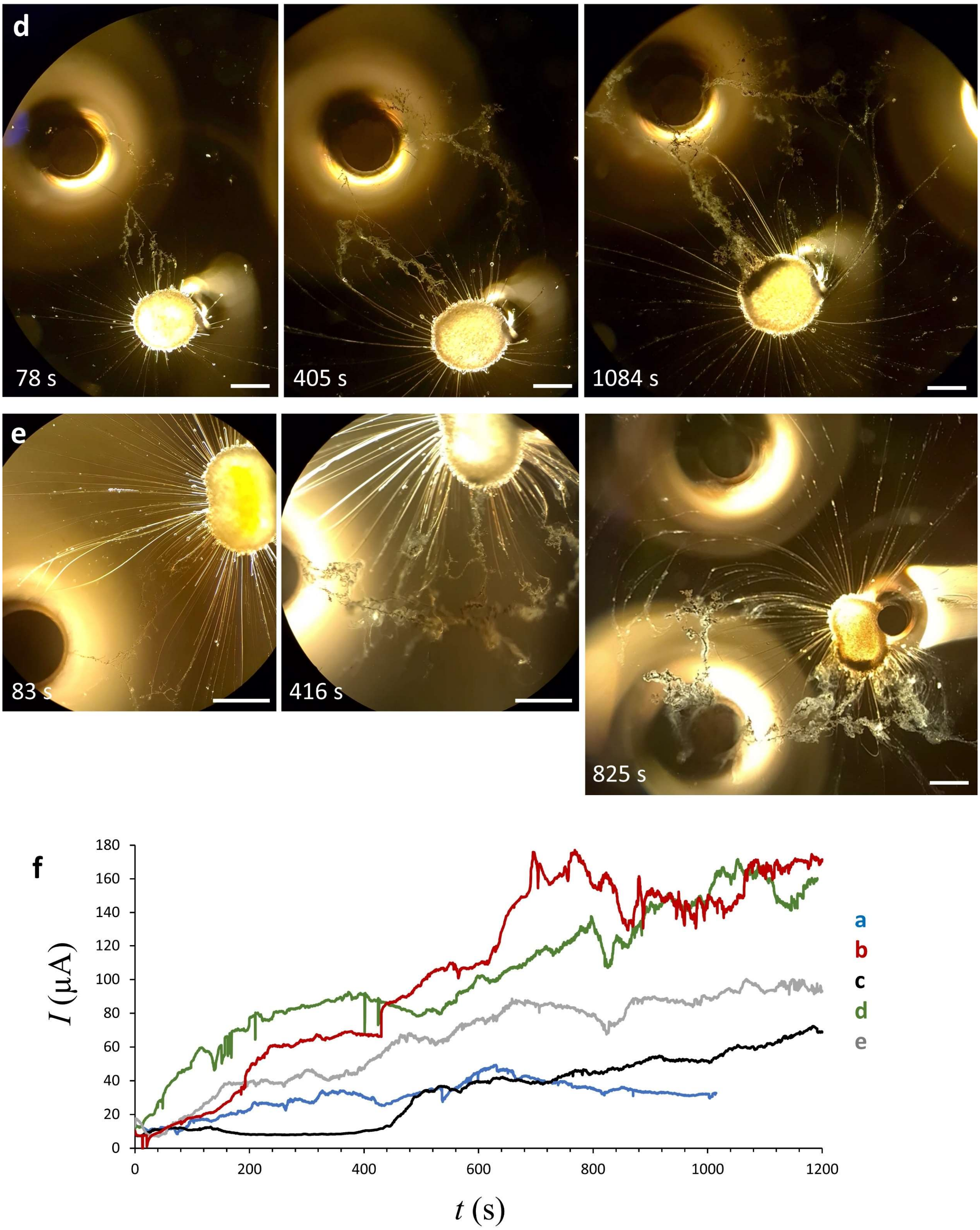

Figure S4 (continued): Optical microscopy recordings on the interaction of copper(II)-loaded myelins and electrodeposited dendrites in five experiments. Similar to the experiment shown in Figure 4 of the main text, a source droplet loaded with $CuCl_2$ (16.7 mol%, 1.0 μL) was deposited on milli-Q water and kept in place by the meniscus of a metal pin in the solution. Next, a voltage of -8 V was applied (t = 0 s). The droplet deposition times are t = -133 s (**a**); t = -155 s (**b**); t = -200 s (**c**); t = -208 s (**d**) and t = -47 s (**e**). The experiments shown in **d** and **e** correspond to the experiments shown in Figure 4 and 5, respectively. The scale bars equal 1 mm. **f)** Current *I* vs time in the myelin-guided electrodeposition of copper(II), corresponding to the experiments shown in **a-e**).

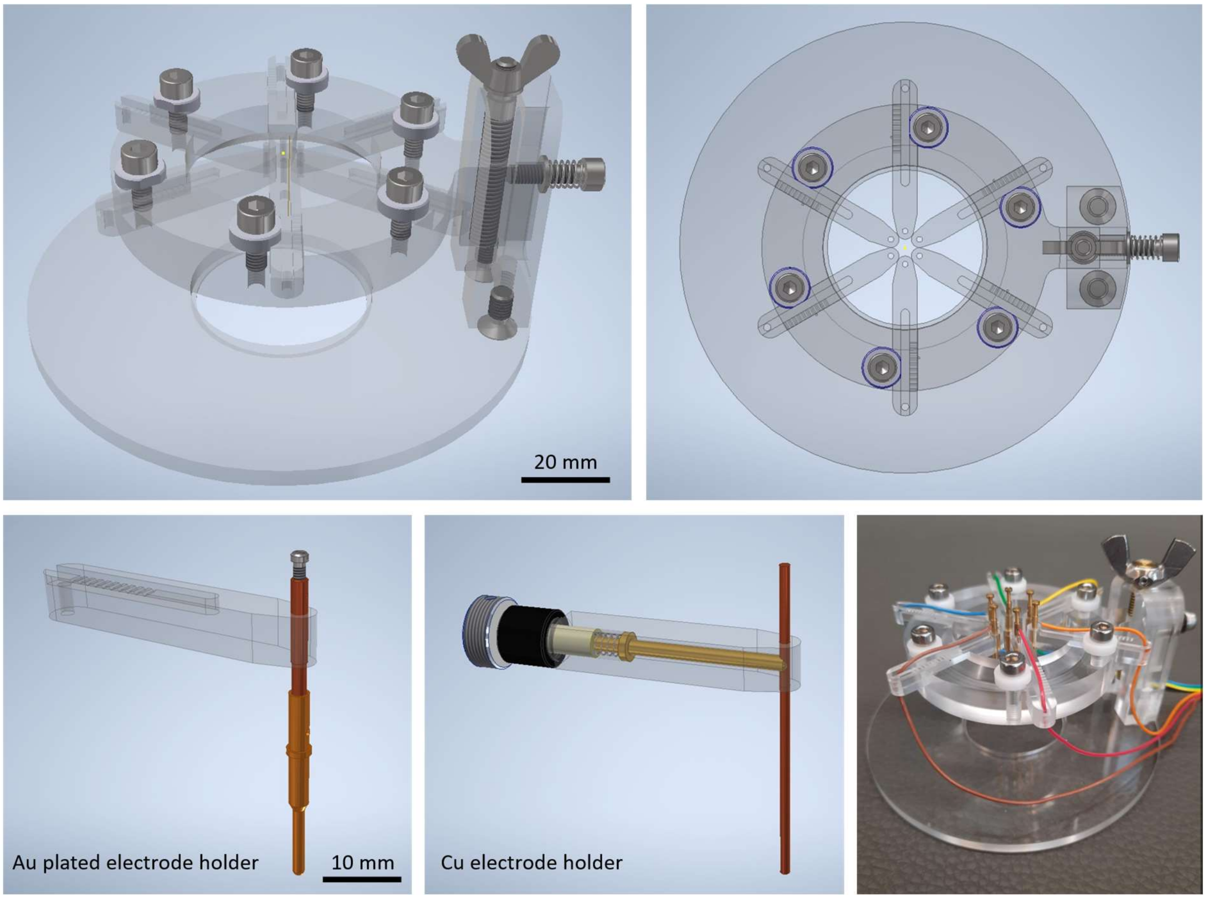

Figure S5: Technical drawings and photograph (bottom right) of the home-made setup that was used to position the electrodes (either gold-plated or copper electrodes) in the solution. The holder was obtained from CNC machined Plexiglas. When placed over a Petri dish, each of the six electrodes in the holder can be positioned closer and further away from the center of the Petri dish. Furthermore, the height of the electrodes can be adjusted. Each electrode was connected to a homemade switch box, such that it could either function as a working electrode, counter electrode or be in the neutral, off state.

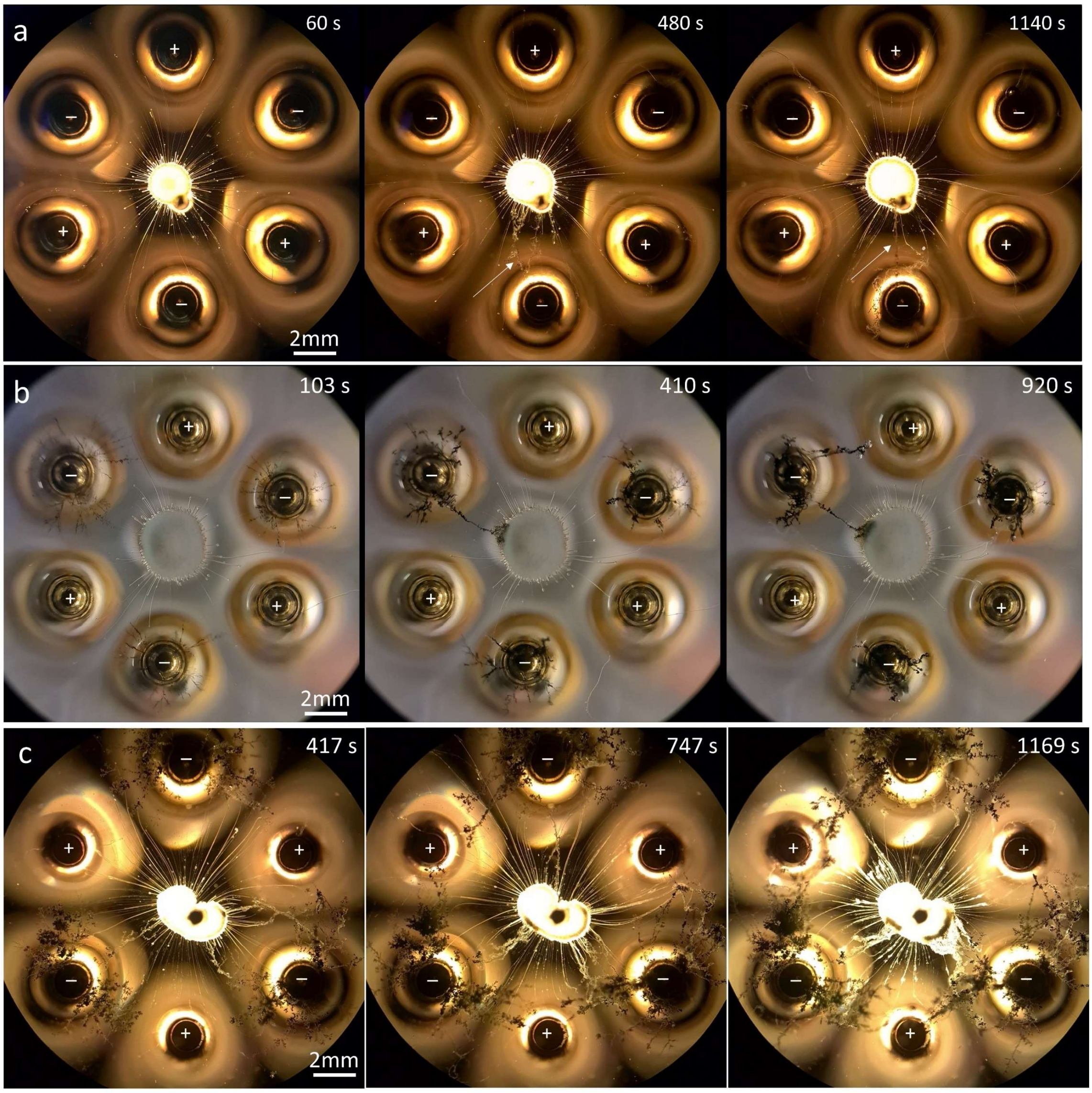

Figure S6: Optical microscopy recordings of copper dendrite electrodeposition amongst $CuCl_2/C_{12}E_3$ source droplets (16.7 mol%, 2.0 µL) that are positioned in between six gold-plated electrodes on milli-Q water **(a)** and a 200 µM $CuCl_2$ solution in water **(b)**. When a potential of -8 V was applied to the three working electrodes, we observe in both experiments that copper dendrites from only one working electrode reach the source droplet (indicated by white arrow in a). Together, these experiments show the limited interaction between the dendrites and 16.7 mol% copper(II)-loaded myelins, which prompted us to use a loading of 18.5 mol% $CuCl_2$ in the experiments where multiple working electrodes are involved. **c)** When a $CuCl_2/C_{12}E_3$ source droplet (16.7 mol%, 2.0 µL) was deposited amongst three gold-plated working electrodes and three copper counter electrodes on a 200 µM $CuCl_2$ solution in water, the electrodeposited dendrites did not establish a connection to the source droplet from all working electrodes.

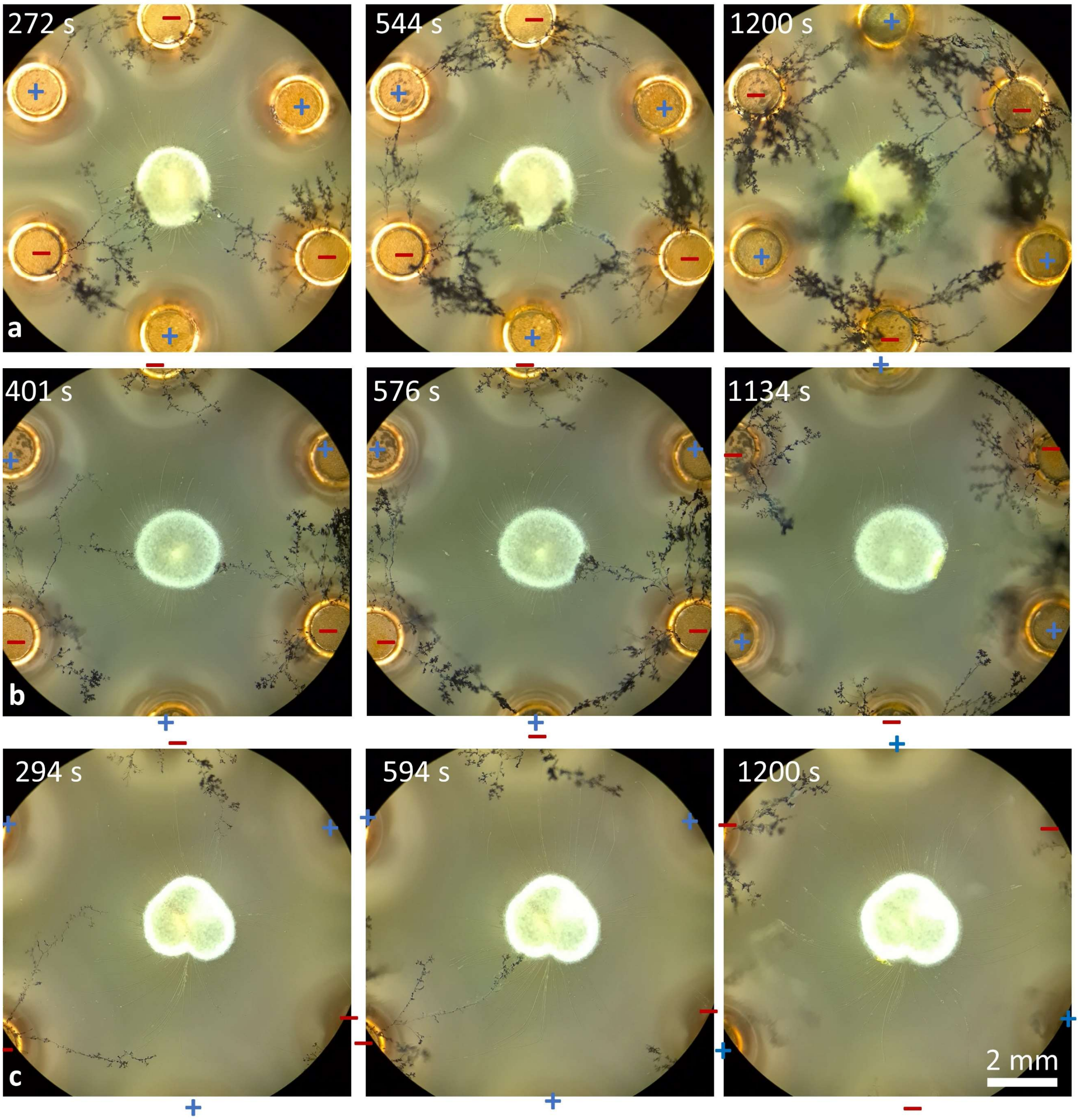

Figure S7 (caption continues on next page)

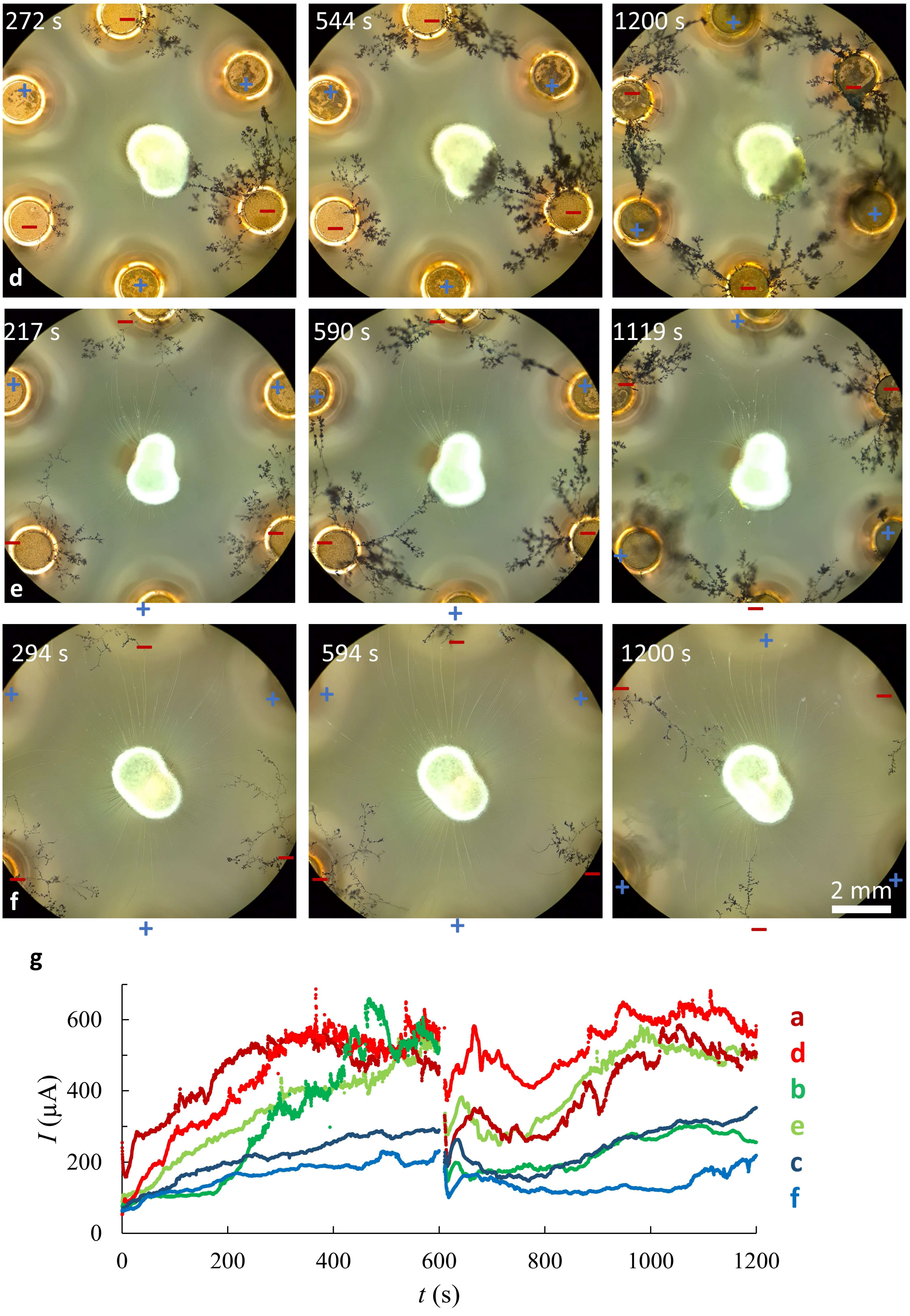

Figure S7: A copper(II)-loaded source droplet (18.5 mol%, 2.0 µL) is positioned amongst six copper electrodes in a hexagonal array on milli-Q water. The source is kept in position via a copper wire inserted in the droplet. The optical microscopy images feature two cycles where the working electrodes (-8 V) and the counter electrode are

switched at t = 600 s. As the potential of the electrodes is switched, we observe the old dendrites to decline, and new dendrites to grow from the three working electrodes while interacting with the copper(II)-loaded myelins, some of which reaching the central source droplet. The effect of the center-to-center distance between the electrodes and source droplet, varying from 4 mm (**a, d**) to 5 mm (**b, e**) and 6 mm (**c, f**) is shown in the optical microscopy recordings. **g)** Current *I* vs time in the myelin-guided electrodeposition of copper(II), corresponding to the experiments shown in **a-f**).

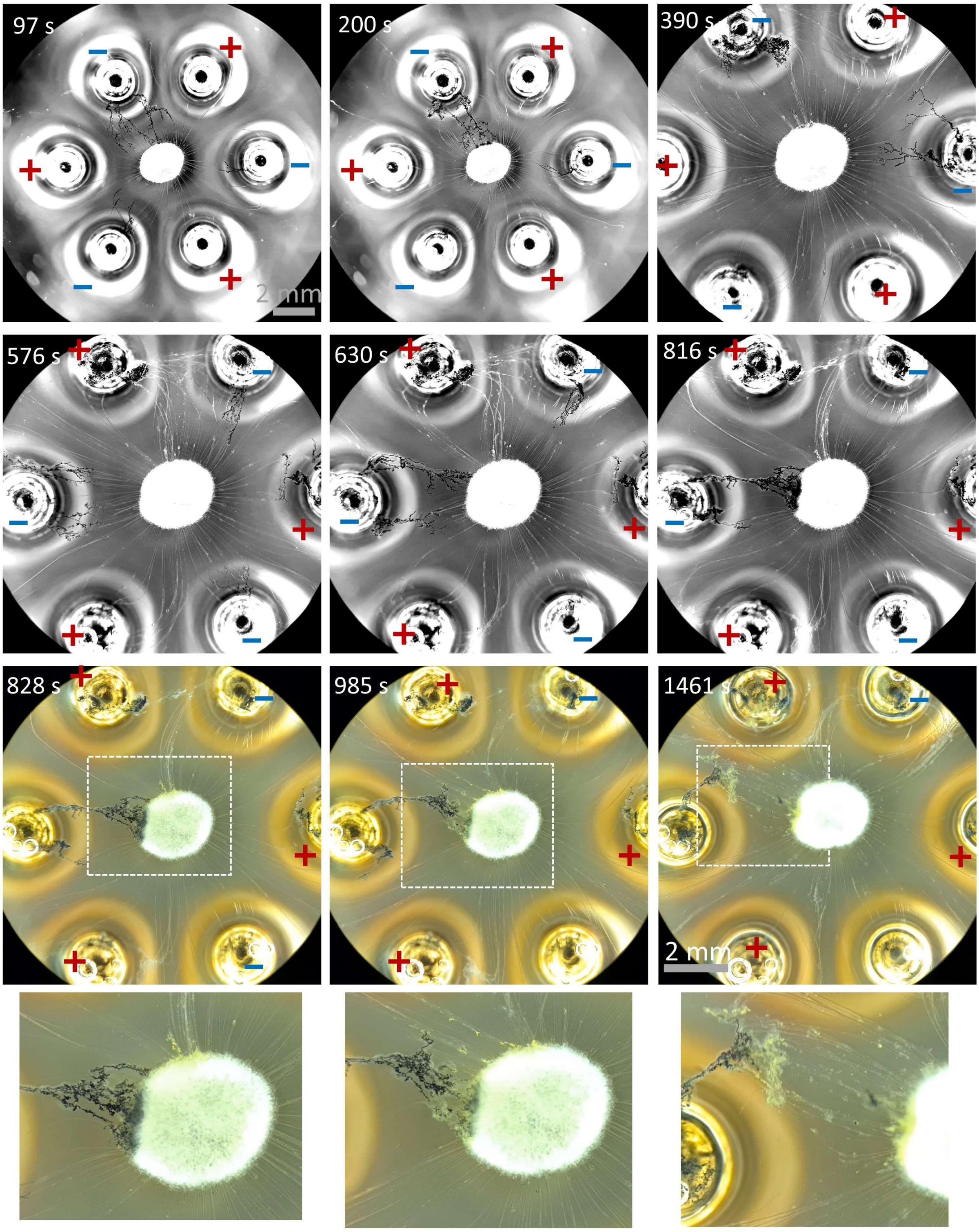

Figure S8: Optical microscopy recordings on the interaction between copper dendrites and copper(II)-loaded myelins growing from a source droplet (2.0 µL, 18.5 mol% $CuCl_2$), positioned amongst six gold-plated electrodes in a hexagonal array on milli-Q water. The source is kept in position via a copper wire inserted in the droplet. A potential of -8 V is applied to the three working electrodes (as indicated in the images), and whereas initially, dendrites grow from all working electrodes (t = 97 s), eventually one electrode dominates as the other dendrites decline (200 s), whereas around t = 390 the system is distorted and another electrode takes over. At t = 509 s, the potential is reversed, and again dendrites growing from one working electrode dominate over the others – featuring the "winner takes all" effect. At the t = 828 s, the potential is turned off from the left and bottom right working electrodes. As a result, the dendrites that grew from the left working electrode detach (as featured in the dashed

white box), and over the course of 10 min, the dendrites turn yellow, indicative of oxidation. The grey images (97 s – 816 s) are converted to grayscale and contrast enhanced, to improve the visibility of the myelins. In the second cycle (t = 509 s), the current was limited to 200 µA (galvanostatic approach) to avoid too much bubble formation.

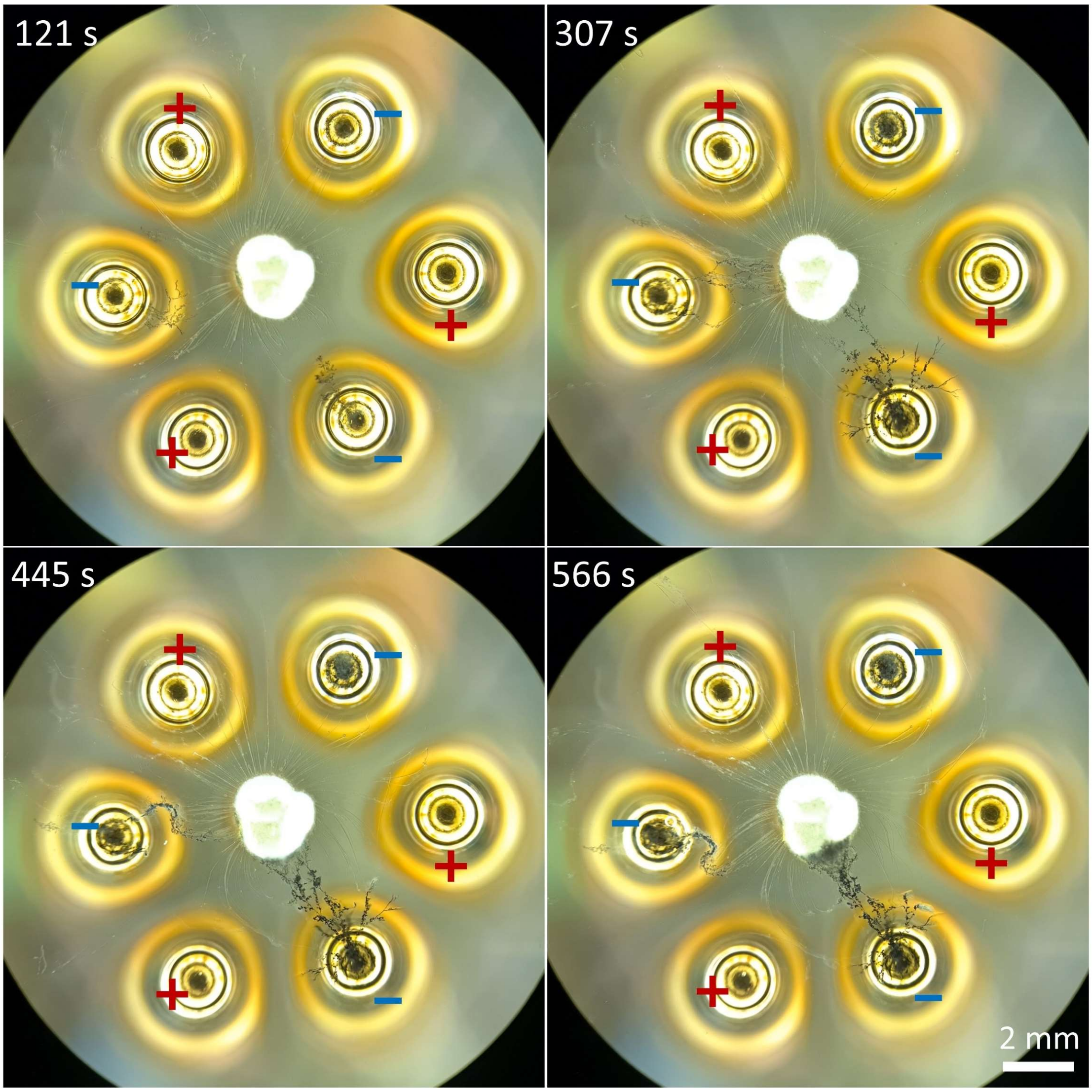

Figure S9: Optical microscopy recordings on the interaction between copper dendrites and copper(II)-loaded myelins growing from a source droplet (2.0 µL, 18.5 mol% $CuCl_2$), positioned amongst six gold-plated electrodes in a hexagonal array on milli-Q water. The source is kept in position via a copper wire inserted in the droplet. A potential of -10 V is applied to the three working electrodes (as indicated in the images). Initially, dendrites grow from the left and bottom right working electrodes (t = 121 s – 307 s); Eventually one electrode dominates as the other dendrites decline (445 s) – featuring the "winner takes all" effect.

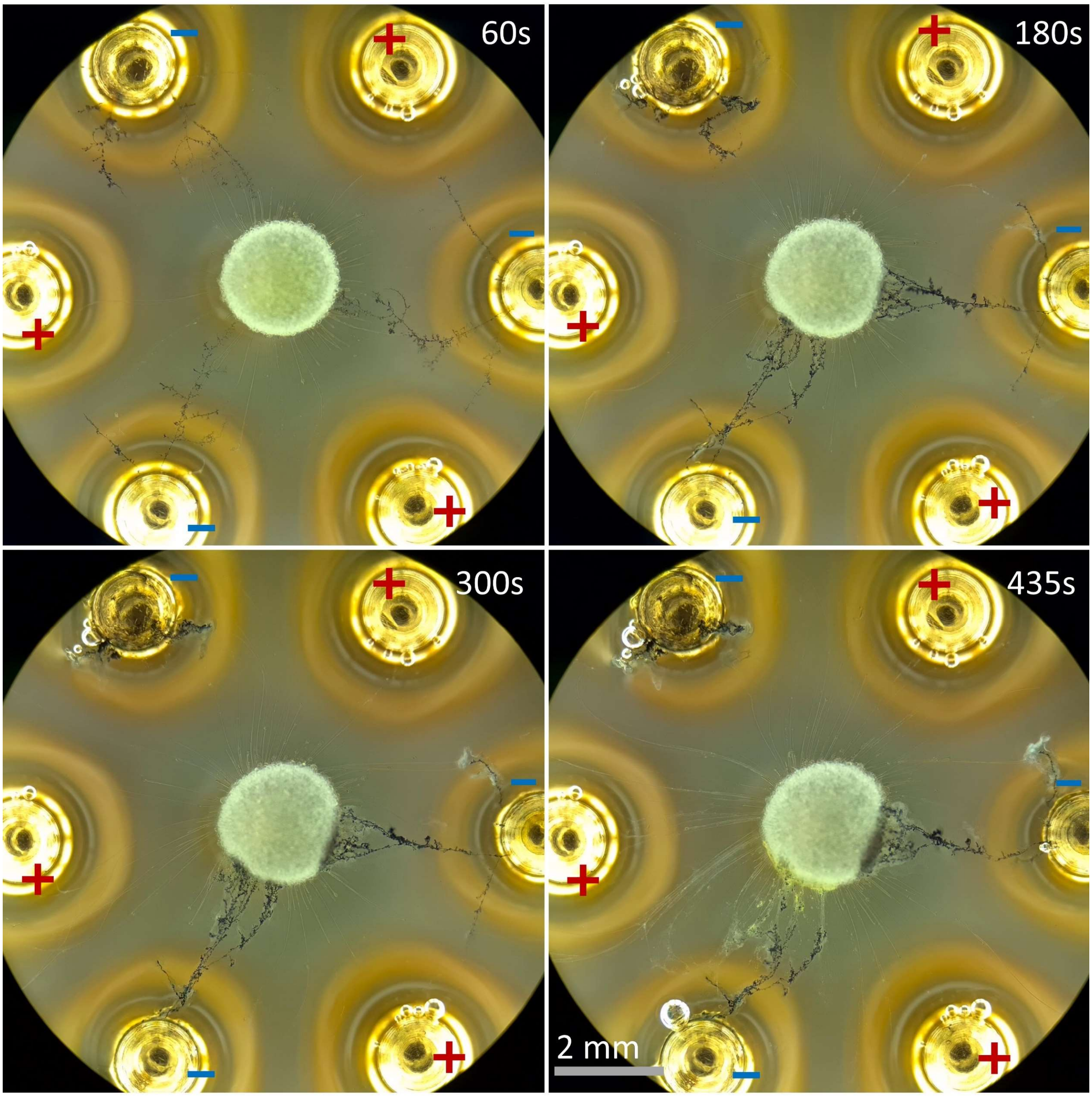

Figure S10: Optical microscopy recordings on the interaction between copper dendrites and copper(II)-loaded myelins growing from a source droplet (2.0 µL, 18.5 mol% $CuCl_2$), positioned amongst six gold-plated electrodes in a hexagonal array on milli-Q water. The source is kept in position via a copper wire inserted in the droplet. A potential of -8 V is applied to the three working electrodes (as indicated in the images). Initially, dendrites grow from all working electrodes (t = 60 s); Eventually one electrode dominates as the other dendrites decline – featuring the "winner takes all" effect.

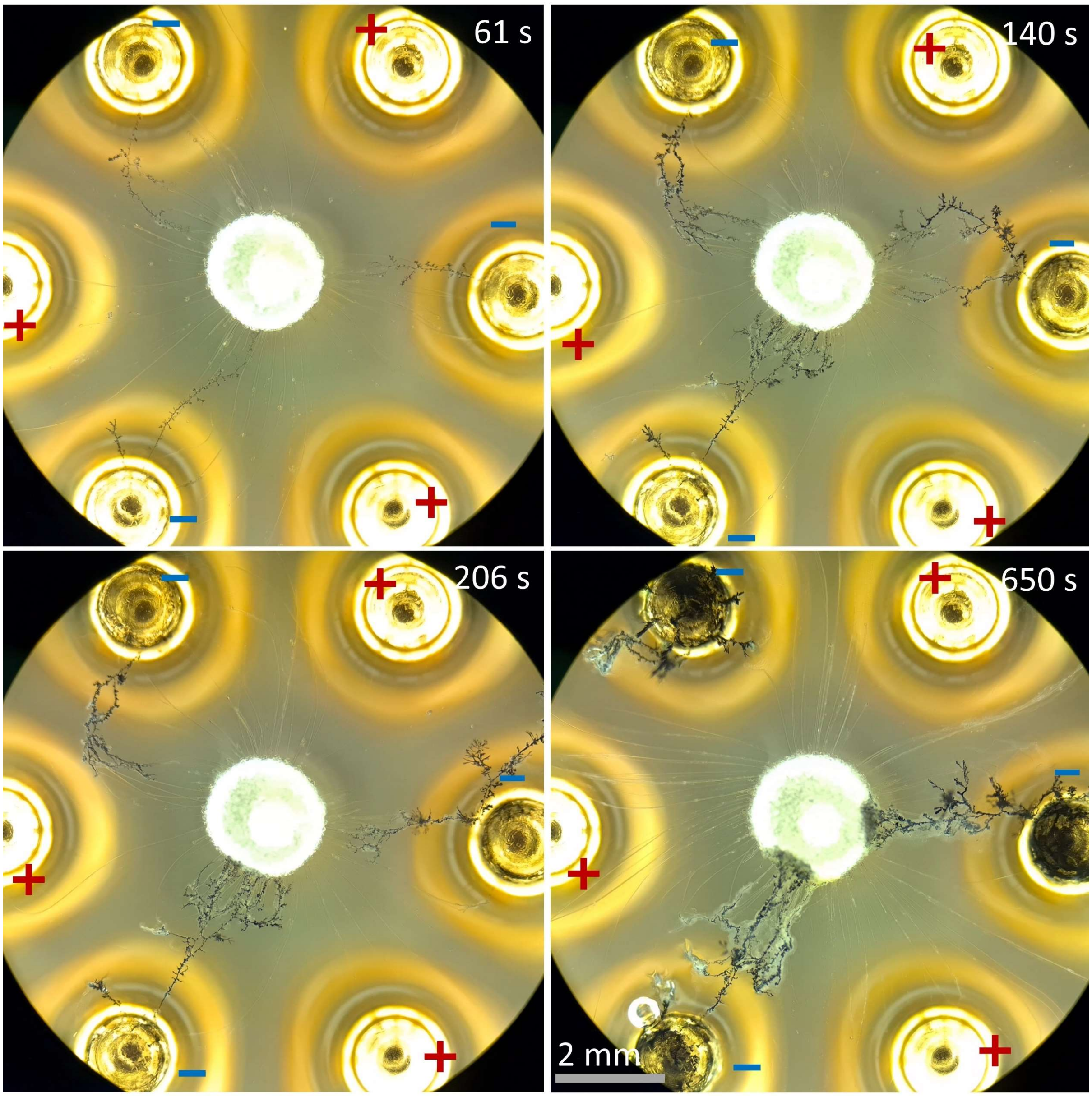

Figure S11: Optical microscopy recordings on the interaction between copper dendrites and copper(II)-loaded myelins growing from a source droplet (2.0 µL, 18.5 mol% $CuCl_2$), positioned amongst six gold-plated electrodes in a hexagonal array on a solution of 100 µM $CuCl_2$ in milli-Q water. The source is kept in position via a copper wire inserted in the droplet. A potential of -6 V is applied to the three working electrodes (as indicated in the images). Initially, dendrites grow from all working electrodes (t = 61 s – 140 s); At t = 206 s, one electrode loses connection to the source, and the other dendrites sustain (650 s).

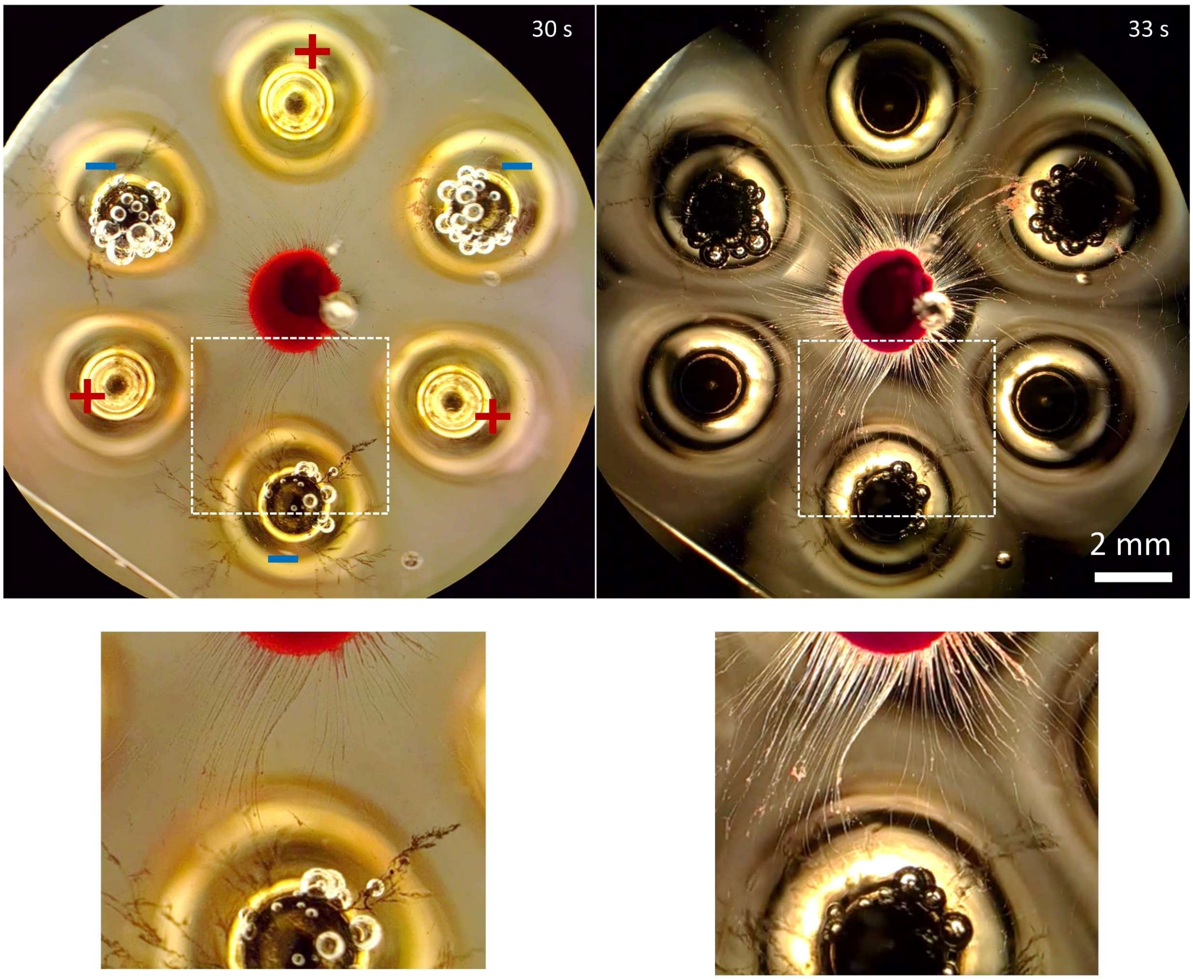

Figure S12: Optical microscopy recordings probing the interaction between copper-free myelins and copper-dendrites. The $C_{12}E_3$ source droplet was loaded with the dye oil red O (20 mg/mL) to enhance the visibility of the myelins in bright field microscopy. The dendrites were electrodeposited from a 200 µM $CuCl_2$ solution in water (-8 V, start at t = 0 s). The source droplet was placed at the meniscus of a metal pin amongst six gold-plated electrodes in a hexagonal array. Both in bright field optical microscopy (t = 30 s) and dark field mode (t = 33 s), no interaction can be observed between the dendrites, which grow under the air-water interface, and the myelins, floating at the air-water interface.

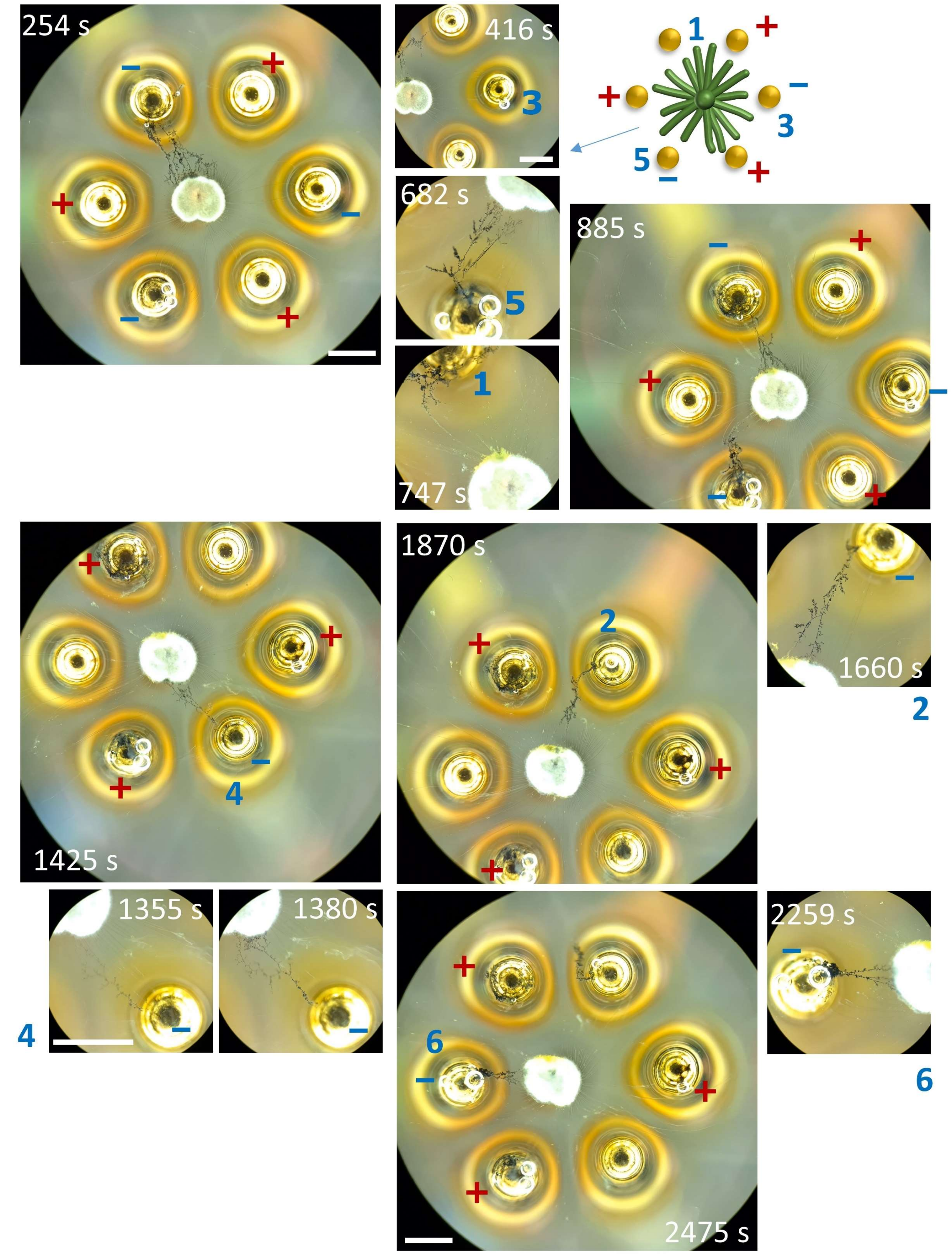

Figure S13: Dynamic, reconfigurable connections between a source droplet (2.0 µL, 18.5 mol% $CuCl_2$) and six gold-plated electrodes, positioned in a hexagonal array around the source droplet. The source is kept in position via a copper wire inserted in the droplet, and the potential is applied to the working electrode(s), such that the current is limited to 200 µA (galvanostatic approach). At t = 254, the potential is applied to three working electrodes, as indicated in the microscopy image, and dendrites grow only from electrode #1 (winner-takes-all principle). At t = 416 s, only electrode #3 and #5 are active as working electrode, and dendrites grow from #5 (682 s), whereas the dendrites from #1 decline (747 s). When #1, #3 and #5 are applied as working electrode together, the dendrites from #5 decline, and #1 dominates (885 s). Next, dendrites are subsequently grown from #4 (1355 s – 1425 s); #2 (1660 s – 1870 s) and #6 (2259 s – 2475 s). The scale bars equal 2 mm.

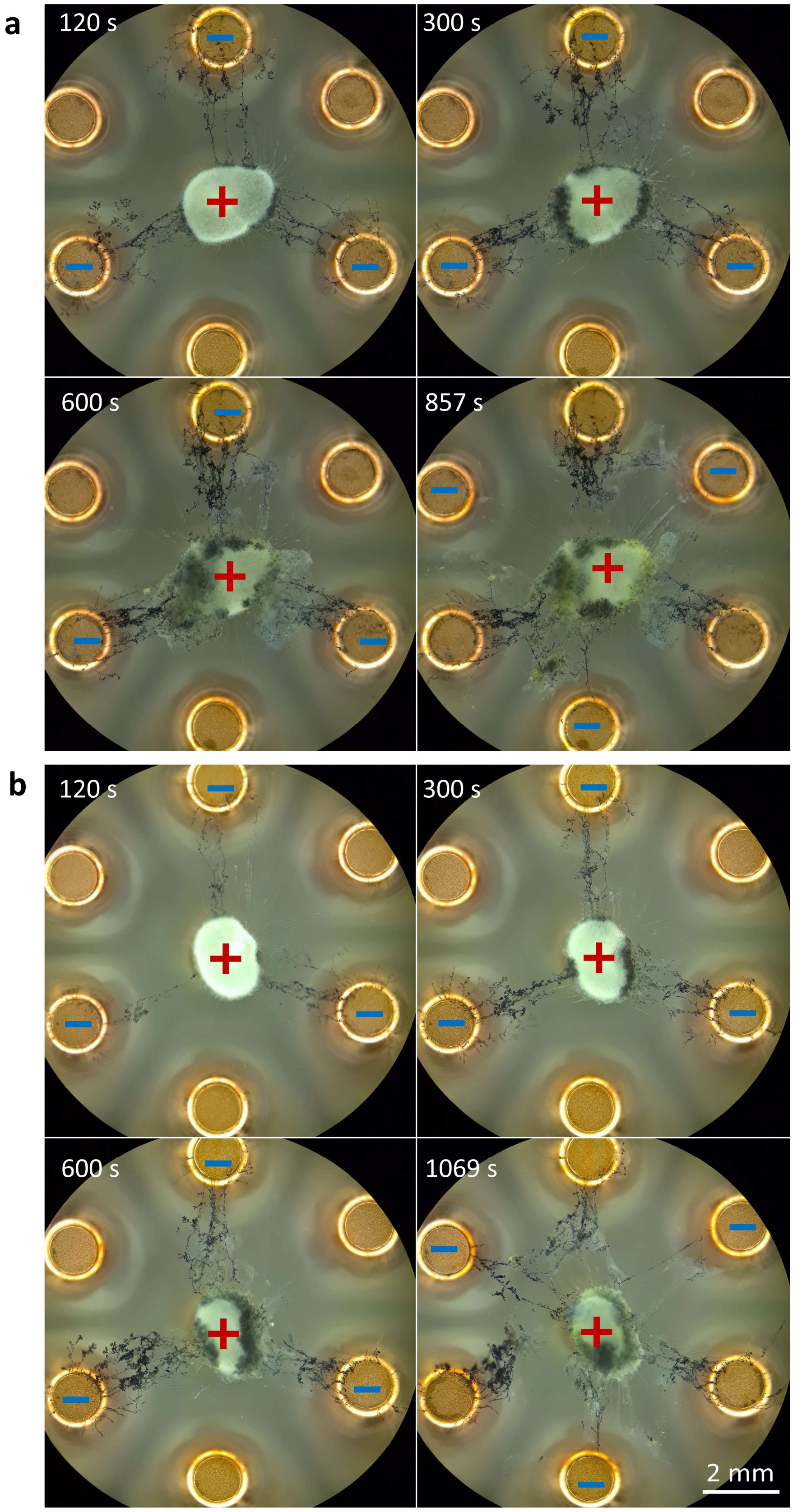

Figure S14: Two experiments (**a,b**) on a copper(II)-loaded source droplet (2.0 µL, 18.5 mol%) positioned amongst six copper electrodes in a hexagonal array on milli-Q water. A copper wire is inserted in the source droplet. When a voltage of -8 V is applied to three working electrodes (t = 0 s), and the copper wire inserted in the source droplet functions as counter electrode, dendrites grow rapidly towards the source. At t = 600, the three other electrodes are applied as working electrode.

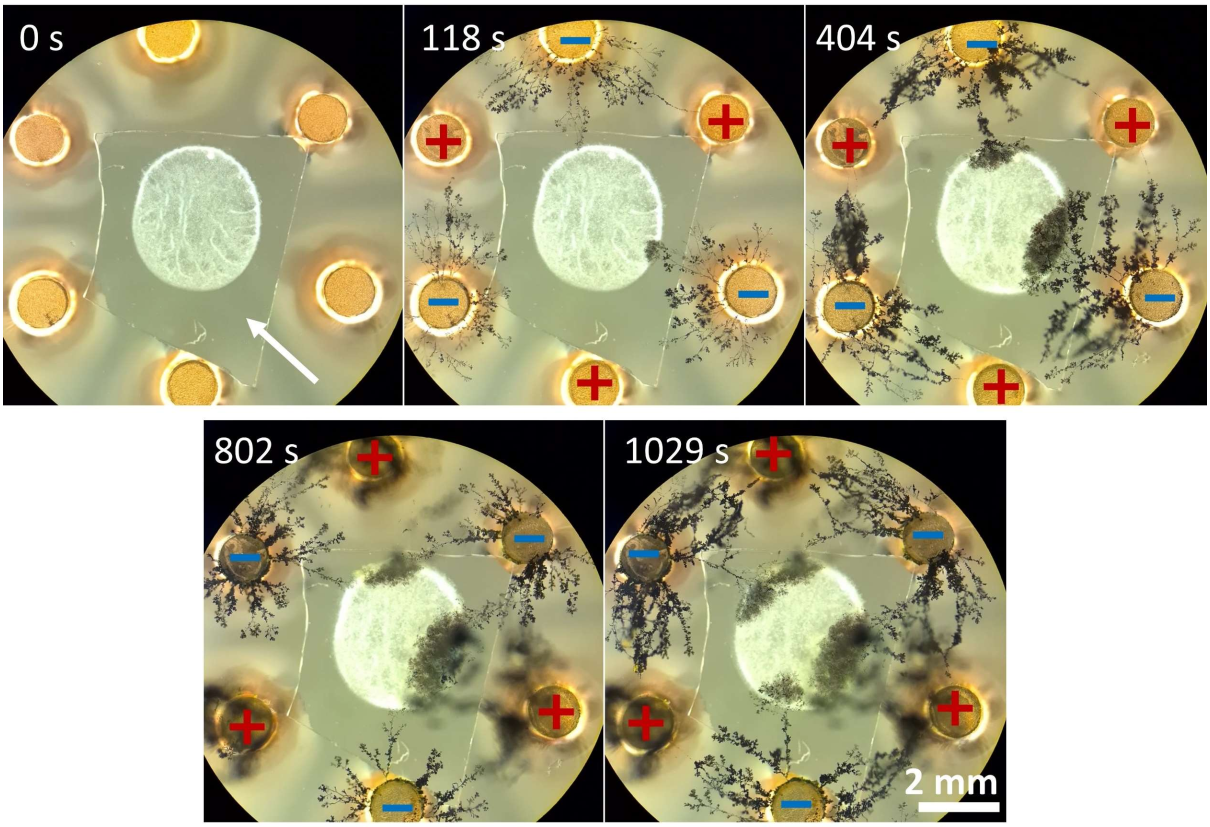

Figure S15: Optical microscopy recordings of a source droplet (1.0 µL, 18.5 mol%) positioned amongst six gold-plated electrodes in a hexagonal array on milli-Q water. To inhibit the growth of myelins, the source droplet is covered with a thin microscopy glass slide at the air-water interface (indicated with white arrow at t = 0 s). At t = 0 s, the potential (-8 V) is applied to three working electrodes; the potential is switched at t = 676 s.

Description of Movies 1 – 6

Movie 1 – corresponding to Figure 2: Optical microscopy recordings of $CuCl_2/C_{12}E_3$ source droplet (16.7 mol%, 1.0 µL) deposited on milli-Q water. Top left: Infusion of water into the $CuCl_2$-loaded source droplet, right after deposition at the air-water interface, displaying a color change from green-yellow to light blue, indicative of copper(II) hydration. While recording the movie, the microscope was focussed alternatingly on the myelins, at the air-water interface, and the features at the bottom and top surface the source droplet, which are slightly above and below the air-water interface, respectively. Top right - Bottom left: The inclusion of $CuCl_2$ affects the myelin structure, generating spheroids at their tips, whereas the thinnest myelins show pearling. Bottom right: Growth of the copper(II)-loaded myelins from a source droplet that has been present at the air-water interface for approx. 20 min.

Movie 2 – corresponding to Figure 3: Optical microscopy recordings of copper dendrite growth from aqueous $CuCl_2$ solutions (10 µM to 1 mM) over a time period of 20 minutes. The movies feature the bottom of the copper working electrode (-8 V) dipped in the $CuCl_2$ solution, the microscope was focussed at the air-water interface to get a sharp image of the growing copper dendrites. The unsharp structures are dendrites that are growing towards the aqueous solution, i.e. away from the air-water interface.

Movie 3 – corresponding to Figure 4: Optical microscopy recordings of copper dendrites growing from the copper working electrode (- 8 V) that interact with the copper(II)-loaded myelins growing from the source ($CuCl_2/C_{12}E_3$ 16.7 mol%, 1.0 µL) on milli-Q water. The movies on the top row show three different stages in the growth process. Top left: Onset of myelin growth from source droplet right after deposition, the potential is applied at $t = 0$ (indicated by timer in the corner), and the dendrites start interacting with the myelins. Top middle: Arrival of dendrites at the source droplet. Top right: Progression of electrodeposition in the source droplet. Bottom: The optical microscopy recordings in the bottom highlight the interaction of the dendrites with the myelins, acquired at larger magnification.

Movie 4 – corresponding to Figure 5: Optical microscopy recordings of the interactions of the copper(II)-loaded myelins (16.7 mol%, 1.0 µL) and the dendrites growing from two different copper electrodes that are consecutively applied as working electrode (-8 V). Top row: In the time window [0 – 1200] s, the bottom electrode is the working electrode (WE), and the three movies show different stages in the growth process. The top right movie features the disconnection of the dendrites and myelins when the voltage was switched (at $t = 1200$ s). Bottom row: In the time window [1200 – 2400] s, the top electrode is the working electrode (WE). The bottom left movie shows the nucleation of dendrites from the electrode; the bottom middle the electrodeposition in the source. The bottom right movie is acquired when the voltage is turned off, showcasing bright field images of the dendrites. We note that the time is indicated in the top right corner of the individual movies, and the voltage was applied at t = 0.

Movie 5 – corresponding to Figure 6: Optical microscopy recordings of multiple connections amongst electrodes and source droplet upon electrodeposition. Left movie: A copper(II)-loaded source droplet ($CuCl_2$, 18.5 mol%, 2.0 µL) is positioned amongst 6 copper electrodes in a hexagonal array on milli-Q water. The source is kept in position via a copper wire inserted in the droplet. The optical microscopy images feature 3 cycles where in an alternating fashion 3 electrodes are the working electrode (-8 V) and 3 electrodes are the counter electrode. As the potential to the electrodes is switched, we observe the old dendrites to decline, and new dendrites to grow from the 3 working electrodes while interacting with the copper(II)-loaded myelins, some of which reaching the central source droplet. Right movie: A copper(II)-loaded source droplet ($CuCl_2$, 18.5 mol%, 2.0 µL) is positioned amongst 6 copper electrodes in a hexagonal array on milli-Q water. A copper wire is inserted in the source droplet. When a voltage

of -8 V is applied to 3 working electrodes, and the copper wire inserted in the source droplet functions as counter electrode, dendrites grow rapidly towards the source.

Movie 6 – corresponding to Figure 7: Optical microscopy recording of dynamic, reconfigurable connections amongst electrodes and source droplet. A copper(II)-loaded source droplet ($CuCl_2$, 18.5 mol%, 2.0 μL) is positioned amongst 6 gold-plated electrodes in a hexagonal array on milli-Q water. The source is kept in position via a copper wire inserted in the droplet. 3 electrodes function as counter electrode, as indicated by the symbols in the movie, and the working electrode alternates from 3 different positions over time. The movie features the dendrites that grow from the active working electrode, while the old dendrites from electrodes that are no longer active decline and get pushed away from the source droplet by the outbound Marangoni flow.
